# Dovetailing the Genetic, Developmental and Functional Bases of the Adaptation of Stomatal Traits to Climate

**DOI:** 10.1111/ele.70453

**Published:** 2026-09-16

**Authors:** Camila D. Medeiros, Thomas N. Buckley, Kevin Sartori, Cyrille Violle, Denis Vile, François Vasseur, Leila R. Fletcher, Matteo Pellegrini, Etienne Baron, Elena Kazakou, Lawren Sack

**Affiliations:** ^1^ Department of Ecology and Evolutionary Biology University of California Los Angeles California USA; ^2^ Department of Botany Federal University of Pernambuco Recife Pernambuco Brazil; ^3^ Department of Plant Sciences University of California Davis California USA; ^4^ CEFE Université Montpellier, CNRS, EPHE, IRD, Université Paul Valéry Montpellier 3 Montpellier Hérault France; ^5^ LEPSE Université Montpellier, INRAE, Institut Agro Montpellier Hérault France; ^6^ Department of Biology Southern Oregon University Ashland Oregon USA; ^7^ Department of Molecular, Cell, & Developmental Biology University of California Los Angeles California USA

**Keywords:** *Arabidopsis thaliana*, climate adaptation, functional traits, GWAS, stomatal development

## Abstract

Stomata, the micro‐valves on leaves, regulate CO_2_ uptake and water loss. Thus, the anatomical maximum stomatal conductance, *g*_max_, is a key influence on gas exchange, growth and productivity. For 145 geographically diverse ecotypes of *Arabidopsis thaliana* grown in a common garden we tested hypotheses for the drivers of variation in *g*_max_ and a stomatal size‐density trade‐off, considering epidermal optimization, epidermal development and climate adaptation. Ecotypes native to colder and drier macroclimates had higher *g*_max_, consistent with leaf‐scale stress‐avoidance, despite having longer times to flowering. A high *g*_max_ was achieved through greater epidermal allocation to stomata, via both increased guard cell initiation and smaller epidermal pavement cells. Stomatal traits showed polygenic associations with climate. A weak stomatal size‐density trade‐off was observed for only the abaxial surface, consistent with flexible constraints on stomatal development and independent trait adaptation. Dovetailing mechanisms influence stomatal adaptation to climate across a wide‐ranging species.

## Graphical abstract

For diverse ecotypes of *Arabidopsis thaliana* grown in a common garden, we tested a three‐tier approach to clarifying the drivers of *g*_max_. Our analyses yielded novel findings for this wide‐ranging species: a larger *g*_max_ was achieved through greater epidermal allocation to stomata, via a higher stomatal index and smaller epidermal pavement cells; larger *g*_max_ is an adaptive phenotype, conferring stress‐avoidance under colder and drier climates; and *g*_max_ shows polygenic associations with climate. Combining genetic, developmental, functional and macroclimate adaptation frameworks highlights new avenues for clarifying the mechanisms and implications of variation in important traits that contribute to ecological adaptation.

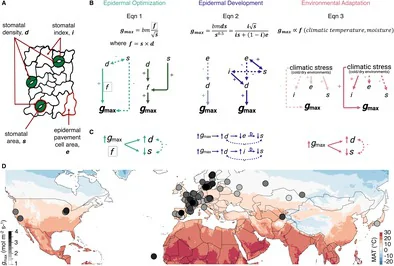

## 1 Introduction

**FIGURE 1:**
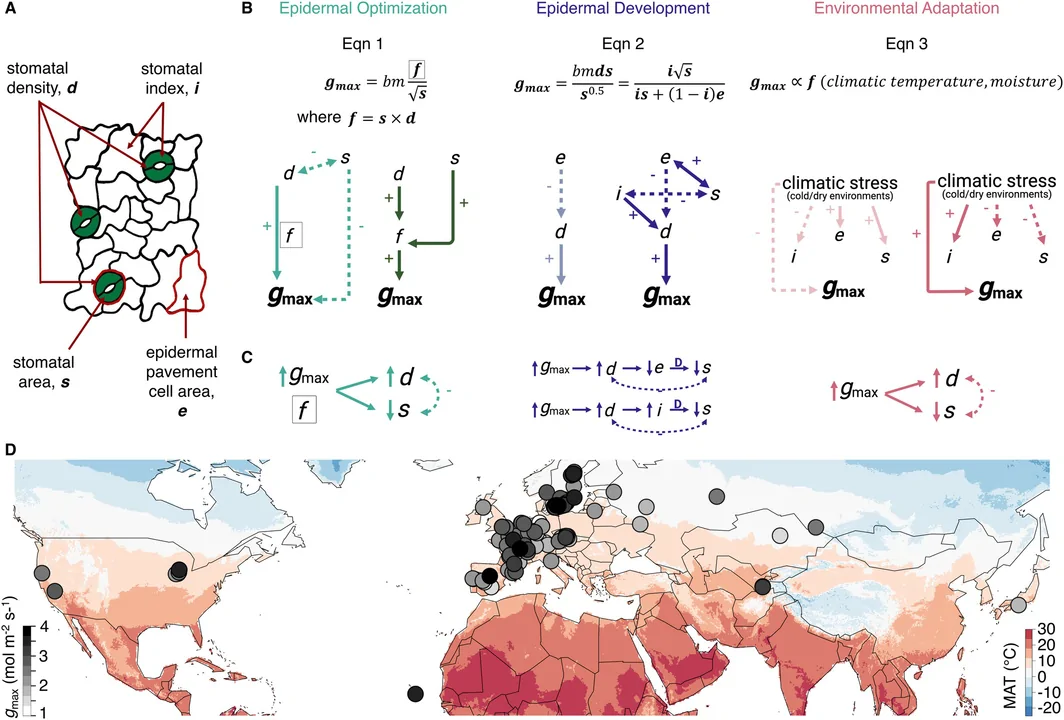
The control of *g*_max_ by environmental adaptation, epidermal development and epidermal optimization. (A) Anatomical traits that determine *g*_max_, stomatal density, *d*, and stomatal area fraction, *f*: epidermal pavement cell area, *e*, stomatal index, *i*, and stomatal size, *s*. (B) The three frameworks for trait determination of the variation of *g*_max_ (Table 1): *Epidermal Optimization* (green), *Epidermal Development* (blue) and *Environmental Adaptation* (pink), including the alternative equations for the calculation of the maximum stomatal conductance, *g*_max_, where *b* is a biophysical constant and *m* a morphological constant based on scaling factors representing the proportionalities of stomatal dimensions (see SI, Equation 4). Solid lines represent positive and dashed lines negative relationships, and colours within each framework represent alternative hypotheses. According to the *Epidermal Optimization* framework, a high *g*_max_ may be driven by either a combination of high *d* and low *s* (minimization of stomatal area fraction, *f*, light green), or by a high *f* driven by high *d* and/or *s* (adaptive *f* hypothesis, dark green). According to the *Epidermal Development* framework, a high *d* could arise from low *e* (passive dilution hypothesis, light blue) or from both a high *i* and/or a low *e* (multiple developmental traits hypothesis, dark blue), resulting in a high *g*_max_. According to the *Environmental Adaptation* framework, high stress may select for lower (stress‐resistance hypothesis, light pink) or higher (stress‐avoidance hypothesis, dark pink) *g*_max_. (C) A *d* versus *s* trade‐off (as shown by a dashed line arrow) could arise from any of the three frameworks, while alternative hypotheses for each framework would suggest weak or absent relationships. According to the minimization of stomatal area fraction hypothesis of the *Epidermal Optimization* framework, a *d* versus *s* trade‐off could arise intrinsically from the evolution of variation in *g*_max_ and simultaneous minimization of *f*, while under the adaptive *f* hypothesis a *s* vs. *d* trade‐off would be weak or nonexistent (not shown). According to the passive dilution hypothesis of the *Epidermal Development* framework, a negative *s* vs. *d* relationship would arise indirectly from a developmental positive association of *e* with *s*, while in the multiple developmental traits hypothesis this trade‐off would arise from either a positive relationship of *e* with *s* or a negative relationship of *i* with *s*; developmental relationships are shown by a “D” above the arrow. According to the stress‐resistance hypothesis of the *Environmental Adaptation* framework, a negative *s* vs. *d* relationship may arise indirectly from a high *d* and *g*_max_ and low *s* being adaptive in warmer and wetter environments, while in the stress‐avoidance hypothesis this relationship would arise from a high *d* and *g*_max_ and low *s* being adaptive in colder, drier macroclimates. (D) The geographic origin of the 145 ecotypes of *Arabidopsis thaliana* included in this study. Symbol colours represent a gradient from low (light grey) to high (dark grey) abaxial *g*_max_.

Stomata evolved in plants that transitioned from aquatic to terrestrial environments between the Silurian and Devonian periods (Chater et al. 2017; Tam et al. 2015; Weiss 1865; Willmer and Fricker 1995), and diversified within and across species over the following more than 400 million years to optimize carbon gain relative to water loss according to their habitat and climate. Stomatal density (*d*; pores m^−2^) and stomatal size (*s*; m^2^ pore^−1^) are among the earliest and most measured plant traits (Dittberner et al. 2018; Doheny‐Adams et al. 2012; Dow, Bergmann, and Berry 2014; Grubb et al. 1975; Hedwig 1793; von Humboldt 1798; Salisbury 1927). The anatomical maximum stomatal conductance (*g*_max_; mol m^−2^ s^−1^) is a key trait often used to capture the contribution of stomata to plant photosynthetic productivity (Figure 1) (Franks et al. 2009; Franks and Beerling 2009; Grubb et al. 1975; Hetherington and Woodward 2003; Taylor et al. 2012). In typical transpiring leaves, the operating stomatal conductance (*g*_op_) is less than *g*_max_, as the pores do not open completely (Dow, Bergmann, and Berry 2014; Franks and Beerling 2009; Franks and Casson 2014; Rudall et al. 2013), yet *g*_op_ approaches *g*_max_ in *Arabidopsis thaliana* genotypes acclimated under favourable conditions (Dow, Berry, and Bergmann 2014), and *g*_max_ correlates within and across species with *g*_op_ and maximum photosynthetic rate (Franks and Beerling 2009; Hetherington and Woodward 2003; Ochoa et al. 2024; Wang et al. 2015). Multiple hypotheses have been generated to explain the association of *g*_max_ with epidermal organization, development and climate [Figure 1 and Table 1, e.g., (Carins Murphy et al. 2016; Franks and Beerling 2009; Sack and Buckley 2016; Zhang et al. 2012)]. Here we simultaneously test integrated genetic, functional, developmental, and ecological bases for *g*_max_ adaptation to climate within the widely distributed model species *Arabidopsis thaliana* L. Heynh (Brassicaceae; hereafter “Arabidopsis”).

**TABLE 1:** Epidermal optimization, epidermal development and environmental adaptation frameworks for the determination of adaptive variation in maximum stomatal conductance (*g*_max_), and their mechanism for a stomatal size–density trade‐off, and the contrasting theories in the literature for each framework, synthesized in this study for analysis of *Arabidopsis* ecotypes native across gradients of cold and aridity. Abbreviations: Eepidermal pavement cell size, *e*; stomatal index, *i*; stomatal area, *s*; stomatal density, *d;* stomatal area fraction, *f*.

Framework	Epidermal optimization		Epidermal development		Environmental adaptation	
Contrasting hypotheses for *g*_max_ adaptive variation	*“f minimization”*	*“f adaptation”*	*“Passive dilution”*	*“Multiple developmental drivers”*	*“Stress‐resistance”*	*“Stress‐avoidance”*
(1) Determination of *g*_max_	*f* is constrained under a stomatal packing limit (0.33–0.5) (de Boer et al. 2016; Muir et al. 2023; Sack and Buckley 2016), since stomata are spaced apart by at least one cell [the “one‐cell spacing rule” (Sachs 1978)], and further constrained by putative selection to provide more surrounding space for stomata to operate independently, to reduce the costs of constructing or operating stomata, and/or to enable greater epidermal allocation to other features, e.g., trichomes (de Boer et al. 2016; Franks and Beerling 2009). High *g*_max_ is achieved with high *d* and small *s*, by shifting to higher values along a *s*‐*d* trade‐off. Thus, a high *g*_max_ for a given *f* would be achieved with a high density of small stomata (de Boer et al. 2016; Franks et al. 2009; Franks and Beerling 2009; Muir et al. 2023), by leveraging the “shallow pore advantage” and lower diffusive resistance of smaller stomata.	High *g*_max_ is achieved with high *f*, and thus with high *d* and possibly a high *s* (Sack et al. 2003).	Variation in *g*_max_ is inversely driven by variation in *e*, with differences in stomatal initiation playing a minor or negligible role (Carins Murphy et al. 2016).	High *g*_max_ is achieved with high *i*, low *e* and/or high *s* (Sack and Buckley 2016).	Low *g*_max_ is adaptive in colder, drier macroclimates (Fletcher et al. 2022; Wang et al. 2015), consistent with stress‐resistance, via reduced maximum gas exchange rates relative to more productive environments (Carlson et al. 2016; Fletcher et al. 2022; Hughes et al. 2017). At the whole‐plant scale, Arabidopsis ecotypes adapted to colder and drier climates are stress‐resistant, with slower growth and longer times to flowering (Exposito‐Alonso 2020; Kazan and Lyons 2016; Sanda et al. 1997).	High *g*_max_ is adaptive in colder, drier macroclimates (Fletcher et al. 2022; Hetherington and Woodward 2003; Liu et al. 2018; Tian et al. 2016; Wilkinson et al. 2001), consistent with stress‐avoidance—i.e., achieving more rapid gas exchange in wetter and warmer periods, to mitigate the shortfall during stressful conditions (Fletcher et al. 2022; Mendes et al. 2022; Oliveira et al. 2017; Teixeira Oliveira et al. 2014; Yin et al. 2020). *A. thaliana* is stress‐avoidant relative to other Arabidopsis species (Bouzid et al. 2019). A previous study of stomatal traits across Arabidopsis ecotypes found a higher *d* in cold, dry macroclimates, consistent with adaptation for stress‐avoidance rather than resistance (Dittberner et al. 2018).
(2) Determination of the association of stomatal size (*s*) and density (*d*)	An *s*‐*d* relationship arises intrinsically from the evolution of variation in *g*_max_ and simultaneous minimization of *f*. This assumed *f* minimization is proposed as the origin of the frequently observed negative across‐species relationship of *s* and *d* (de Boer et al. 2016; Franks et al. 2009).	*s* vs. *d* trade‐off may be weak or nonexistent.	Given *e* can be a negative developmental driver of *d*, a negative *s* vs. *d* relationship would arise indirectly from a developmental positive association of *e* with *s* (cell size coordination; Box S1A).	Given *e* and *i* may be, respectively, negative and positive developmental drivers of *d* (Sack and Buckley 2016), a negative *s* vs. *d* relationship would arise indirectly from a positive relationship of *e* with *s* (cell size coordination; Box S1A). Or, a negative of *i* with *s* (cell size coordination or iterative differentiation; Box S1B).	A negative *s*‐*d* relationship may arise indirectly from a high *d* and *g*_max_ being adaptive in warmer and wetter environments, and also a smaller *s*, would provide a competitive benefit due to more rapid opening and closing responses and thus, potentially, a higher light‐ and/or water‐use efficiency (Aasamaa and Sober 2011; Henry et al. 2019; Lawson and Blatt 2014; Medeiros et al. 2019).	A negative *s*‐*d* relationship may arise indirectly from a high *d* and *g*_max_ being adaptive in colder, drier macroclimates, and also a smaller *s*, to provide more rapid opening and closing responses for optimal function when conditions are favourable (Aasamaa and Sober 2011; Henry et al. 2019; Lawson and Blatt 2014; Medeiros et al. 2019).

Previously, variation in *g*_max_ within and across species has been explained within three typically separate frameworks (Table 1; Figure 1B). First, the *epidermal optimization* framework (Table 1; Figure 1B) focuses on the importance of a high *g*_max_ relative to the allocation of epidermal space to stomata, that is, the stomatal area fraction, *f* (= *s* × *d*) (Figure 1B, Equation 1). According to a well‐recognized “*f*‐minimization” hypothesis, *f* is constrained and a higher *g*_max_ evolves to achieve higher gas exchange capacity relative to cost, by shifting along an intrinsic *s*‐*d* “trade‐off” to higher *d* and smaller *s* values (de Boer et al. 2016; Doheny‐Adams et al. 2012; Franks et al. 2009; Franks and Beerling 2009) (further explanation in Table 1; Figure 1B). Yet, a role for constrained *f* in the adaptation of *g*_max_ and the universality of an *s* vs. *d* relationship has not been consistently supported; in some studies within and across species, a higher *g*_max_ did not correspond to a constrained *f* or to a lower *s*, but rather to higher *f* and similar or higher *s* (Dow, Bergmann, and Berry 2014; Liu et al. 2018; Ochoa et al. 2024; Sack et al. 2006; Taylor et al. 2012) (Table S1). These findings suggested a second, “adaptive *f*” hypothesis, where the benefit of a higher *g*_max_ outweighs its putative spatial costs, such that a higher *g*_max_ may evolve from an increase in *f* (Liu et al. 2021) (Table 1; Figure 1B).

A second framework explaining *g*_max_ variation is based on *epidermal development* (Table 1; Figure 1B) and has also generated multiple partially‐contrasting hypotheses. According to the “passive dilution” hypothesis, higher *g*_max_ can result developmentally from smaller mean epidermal pavement cell size (*e*), which would reduce the separation of stomata and thus drive a higher *d* in the mature leaf (Figure 1B, Equation 2) (Baird et al. 2024; Carins Murphy et al. 2014; Sack and Buckley 2016; Salisbury 1927), a mechanism supported for Arabidopsis Col‐0 grown under different irradiances, but not across a set of stomatal mutants (Carins Murphy et al. 2017). According to the “multiple developmental drivers” hypothesis, higher *g*_max_ is also importantly driven by a higher stomatal index [*i*, the proportion of epidermal cells that differentiate into pairs of guard cells (Berger and Altmann 2000; Bergmann and Sack 2007; Geisler and Sack 2002; Salisbury 1927)] and, much less, by a smaller mean *s* (Baird et al. 2024; Sack and Buckley 2016) (Table 1; Figure 1B).

Finally, the *environmental adaptation* framework (Table 1; Figure 1B) considers the variation in *g*_max_ related to external conditions, especially macroclimate, given that *g*_max_ would evolve under selection for higher productivity (Figure 1B, Equation 3) (Sack et al. 2003). Contrasting hypotheses exist for how *g*_max_ would generally adapt under stressful climates, i.e., via “stress‐resistance” or “stress‐avoidance” [terminology after (Fletcher et al. 2022)]. Adaptation to stressful conditions via *resistance* would entail a lower *g*_max_ for greater water use‐efficiency and/or water retention in plant and soil, whereas stress*‐avoidance* would entail a higher *g*_max_, for rapid gas exchange during periods of high resource availability (Table 1; Figure 1B). Arabidopsis ecotypes are known to adapt to colder habitats via stress‐resistance at the scale of the plant life cycle, with slower overall growth and longer times to flowering (Exposito‐Alonso 2020; Kazan and Lyons 2016; Sanda et al. 1997), yet stress‐avoidance may be a stronger mechanism for adaptation at leaf scale (Grubb 1998). Theory for environmental adaptation has also predicted a higher and more variable *g*_max_ for the abaxial (lower) than the adaxial (upper) leaf surface, as shifts to higher adaxial *d* may increase the risks of photodamage to guard cell chloroplasts, of water blockage of pores and infection by pathogens (Muir 2015; Muir et al. 2023).

Synthesizing and simultaneously testing hypotheses from the three frameworks for explaining *g*_max_ variation can provide novel resolution of the coordinated adaptation of gas exchange and stress tolerance, and novel avenues for modulating gas exchange capacity through breeding (Buckley et al. 2020; Caine et al. 2019, 2023; Hughes et al. 2017; Lawson and Leakey 2024; Tanaka et al. 2013).

In addition to their application to *g*_max_, these three frameworks provide non‐exclusive explanations for the frequently reported *s*‐*d* trade‐off across species sets (explained in Table 1, row 2; Figure 1C; studies compiled in Table S1). According to the epidermal optimization framework, the *s*‐*d* trade‐off arises from *f* minimization (de Boer et al. 2016; Franks et al. 2009). According to the developmental framework, the *s*‐*d* trade‐off would arise from tight coordination of guard cell and epidermal pavement cell expansion (i.e., a positive correlation of *e* and *s*), and/or to a trade‐off between *i* and *s* (Sack and Buckley 2016), relationships expected from developmental constraints (Box S1). According to the environmental adaptation framework, the *s*‐*d* trade‐off can arise due to independent selection of a high *d* and low *s* by environmental drivers (Medeiros et al. 2019).

Here, we provide a new synthesis of the three frameworks to test the drivers of variation in *g*_max_ and of the *s*‐*d* trade‐off, and their genetic basis. We focused on 145 ecotypes of Arabidopsis, an annual herb widely distributed across the Northern hemisphere with populations adapted to contrasting macroclimates (“ecotypes”; Figure 1D) (Hoffmann 2002; Sharbel et al. 2000; Weigel and Mott 2009), grown in a greenhouse common garden. Arabidopsis is well established as an excellent system to study physiology, development and ecology within an evolutionary framework (Alonso‐Blanco et al. 2016; Mitchell‐Olds and Schmitt 2006; Sharbel et al. 2000).

## 2 Material and Methods

### 2.1 Plant Material and Growing Conditions

We grew 145 ecotypes of Arabidopsis from the 1001 Genomes Consortium populations (Alonso‐Blanco et al. 2016) selected to represent the extent of the natural distribution of the species (Figure 1D), and the RegMap populations (Horton et al. 2012) in a common greenhouse (Supporting Information, “Plant growing conditions”). This common garden approach is a powerful and frequently used design that can address questions of adaptation in the sense of trait‐climate association that is due to heritable phenotypic variation, as it minimizes confounding environmental variables that may elicit plastic trait variation if plants were sampled in the field (Baird et al. 2021; Huxman et al. 2022). Notably, this approach is subject to specific assumptions if used to support narrower definitions of adaptation, i.e., that the phenotype provides a home‐site fitness advantage (Appendix S3; Tables S8–10).

### 2.2 Trait Measurements

For all trait measurements, for each of three individuals per ecotype, a fully expanded, light‐exposed leaf, or, for bolting individuals, the last developed leaf, was fixed in formalin–acetic acid–alcohol solution (FAA) for at least two days and then transferred to pure glycerol for storage, and traits were measured manually on micrographs taken of nail varnish impressions of both leaf surfaces, using the software ImageJ (Schneider et al. 2012) (Supporting Information, “*Image analysis*”). We measured stomatal density (*d*), stomatal area (*s*), and epidermal pavement cell area (*e*) of the abaxial and adaxial surfaces, and calculated the stomatal index (*i*, the number of stomata divided by the sum of epidermal pavement cells and stomata), the maximum theoretical stomatal conductance (*g*_max_) and the stomatal area fraction (*f*) (Medeiros et al. 2019; Sack et al. 2006). To determine whole leaf‐level trait values, for cell dimensions, we calculated the arithmetic mean of the abaxial and adaxial values (subscript “mean”), and for cell densities and *g*_max_ we calculated the sum of abaxial and adaxial values (subscript “total”).

### 2.3 Macroclimate of Ecotypes' Native Distributions and Flowering Time

We obtained ecotype collection coordinates from the 1001 Genomes Consortium (Alonso‐Blanco et al. 2016) and RegMap panel (Horton et al. 2012) and used R software [version 3.4.4 (R Core Team 2020)] to extract six salient macroclimate variables included in previous studies of Arabidopsis that considered the linkage of traits with cold and/or dry climate ecotypes (e.g., Dittberner et al. 2018; Fletcher et al. 2022; Lasky et al. 2012; Sartori et al. 2019): mean annual aridity index (for which lower values represent more arid climates), annual and growing season mean temperature and precipitation, and the length of the growing season (Supporting Information, “Extraction of macroclimate variables”).

We also obtained the flowering time at 16°C (*F*_16_) for each of the 137 ecotypes included in the 1001 Genomes Consortium (Alonso‐Blanco et al. 2016).

### 2.4 Statistical and Comparative Analyses

All statistical analyses were performed and plots created using R software [version 4.2.1; (R Core Team 2022)] and packages available from the CRAN platform (see Supporting Information, “Statistical and comparative analyses” for more details).

To quantify the trait variation across ecotypes, we performed ANOVAs (Hothorn et al. 2008; Sokal and Rohlf 2012). To test for differences in traits between the abaxial and adaxial surfaces, we performed paired *t*‐tests (Sokal and Rohlf 2012). We also calculated parametric and non‐parametric coefficients of variation of anatomical traits across ecotypes.

To test the relationships among traits and the macroclimate of the origin of each ecotype, we performed ordinary least squares regression (OLS) tests for both untransformed and log_10_‐transformed data, to test for either approximately linear or non‐linear (i.e., approximate power‐law) relationships, respectively. Further, to account for the lack of phylogenetic independence among the ecotypes, we performed generalized mixed‐effects regression analyses, including a kinship matrix as a random effect, using functions available in the ‘coxme’ package [version 2.2.18; (Yu et al. 2006)]. We only considered as significant the relationships for which *p* ≤ 0.05 in both analyses and report the OLS results of the strongest patterns in the main text (Table S6).

To determine the sensitivity of *g*_max_ to leaf anatomical parameters *e*, *i* and *s*, and of *g*_max_ to *f* and *s*, we performed “intrinsic” and “realized” sensitivity analyses of Equations 1 and 2 (Table S5; Figure 1B) (John et al. 2017). In the intrinsic sensitivity analysis, we computed sensitivity coefficients for each input, i.e., by what proportion would *g*_max_ change for an infinitesimal increase in a given input, with all others held constant at their means, e.g., ∂ln[*g*_max_]/∂ln[*s*] = [*s*/*g*_max_]⋅∂*g*_max_/∂*s*. In the realized sensitivity analysis, we conducted causal partitioning analyses of the variation in *g*_max_ contributed by the underlying variables considering the actual background of variation in all variables (Buckley and Diaz‐Espejo 2015) (Table S5). Each contribution is expressed relative to the sum of all contributions (*C*), such that contributions add up to 100%. We used this method to partition: (i) abaxial (and adaxial) *g*_max_ into contributions from abaxial (and adaxial) values of *e*, *i* and *s*, and of *f* and *s*, (ii) total *g*_max_ into contributions from abaxial and adaxial *g*_max_, and (iii) total *g*_max_ into contributions from both abaxial and adaxial values of *e*, *i* and *s*, and of *f* and *s*.

### 2.5 Genetic Analyses

For each trait, we estimated the narrow‐sense trait heritability, i.e., the proportion of phenotypic variance explained by ecotype (*p*_ve_) (Atwell et al. 2010; Clo et al. 2019). The narrow‐sense heritability estimates are applicable to the conditions of the tested common garden. We used a single nucleotide polymorphism (SNP) dataset to conduct a classical genome‐wide association study (GWAS) to test associations of SNPs with anatomical traits (*g*_max_, *d*, *f*, *e*, *i* and *s*) and key macroclimate variables of ecotype origins and to perform a polygenic GWAS (Bayesian sparse linear mixed‐model, BSLMM) to estimate SNP effects on each variable. Additionally, we used the top 1% SNPs (0.5% strongest negative and 0.5% strongest positive) to find the stomatal traits with weak and/or high statistical enrichment of genes related to molecular functions and biological processes (Supporting Information, “*Genetic analyses*”).

## 3 Results

### 3.1 Variation in Leaf Epidermal Traits Across Ecotypes and Its Genetic Basis

**FIGURE 2:**
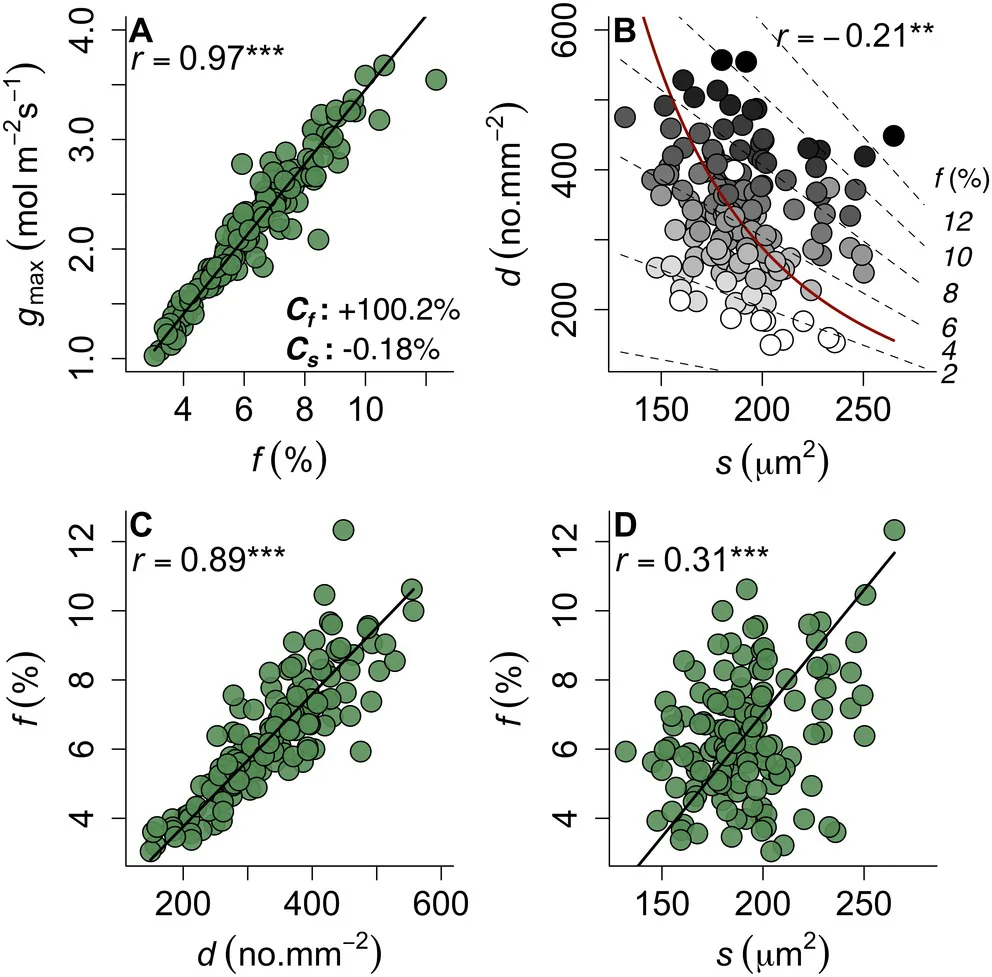
Epidermal optimization of *g*_max_. Coordination of functional drivers of maximum stomatal conductance, *g*_max_, for the abaxial surface across 145 ecotypes of *Arabidopsis thaliana* from diverse geographical origins grown under greenhouse conditions. (A) Relationship of *g*_max_ with stomatal area fraction, *f*. The causal contribution, *C*, is the median contribution (%) of differences in *f* and *s* to differences in anatomical leaf *g*_max_ in the abaxial surface. Medians were calculated over all possible pairwise comparisons between ecotypes (*n* = 10,440 comparisons). (B) Relationship of stomatal density, *d*, with the stomatal size, *s*, with symbol colours representing a gradient from low (light grey) to high (dark grey) *g*_max_, to allow the visualization of the role of variation in *f* in the determination of *g*_max_. Dashed lines represent the *d* vs. *s* curves at five different constant *f*s. Relationships of *f* with (C) *d* and (D) *s*. Solid lines represent the standardized major axis fit. **p < 0.01; ***p < 0.001.

Across the 145 Arabidopsis ecotypes, leaves varied strongly in all measured traits (*p* < 0.001; Table S3). Most of the total trait variance was explained by differences among ecotypes rather than within ecotypes (64 and 36%, respectively; Table S3). Ecotypes varied by two‐ to eight‐fold in maximum anatomical stomatal conductance (*g*_max_), stomatal density (*d*), stomatal area fraction (*f*), epidermal pavement cell area (*e*), stomatal index (*i*) and stomatal area (*s*), for each of the abaxial and adaxial leaf surfaces and when considering them together (i.e., summing for *d* and *g*_max_, and averaging for *i*, *e* and *f*; Table S3). On average, *g*_max_, *f* and *d* were higher for the abaxial surface, *e* was higher for the adaxial surface, *i* and *s* were statistically similar for the two surfaces, and the *g*_max_, *d* and *f* showed stronger variation on the abaxial leaf surface, whereas *e*, *i* and *s* varied similarly on both surfaces (*p* < 0.05; Figure S1, Table S4). The variation across ecotypes in total *g*_max_ was driven by the variation in *g*_max_ on both surfaces (43.2% and 53.7% by abaxial and adaxial surfaces, respectively; Table S5). Across Arabidopsis ecotypes, *s* and *d* were associated for only the abaxial surface (*r* = −0.21; *p* < 0.01; Table S6; Figure 2B), and statistically independent on the adaxial surface, and considering both surfaces combined (Table S6).

Stomatal traits showed strong genetic control across ecotypes, as indicated by high estimates of narrow‐sense trait heritability for all traits except *i* on the adaxial surface (Table 2). Notably, genome‐wide association tests did not identify any significant associations in coding regions between individual stomatal traits and the 1,709,387 single nucleotide polymorphisms (SNPs) tested (Figures S2–S3). When focusing on the top‐1% SNPs (representing the strongest 0.5% positive and 0.5% negative trait associations), several stomatal traits showed modest enrichment for genes implicated in diverse cellular and biological processes and molecular functions, notably those related to cell division and developmental regulation (Table S2).

**TABLE 2:** Narrow‐sense heritabilities for the developmental traits of 145 ecotypes of *Arabidopsis thaliana* with available genome data grown under greenhouse conditions.

Trait	Heritability [*p*_ve_ (SE)]		
	Abaxial	Adaxial	Total
Maximum stomatal conductance (*g*_max_)	0.44 (0.17)	0.59 (0.12)	0.57 (0.12)
Stomatal density (*d*)	0.45 (0.16)	0.63 (0.12)	0.59 (0.12)
Epidermal pavement cell area (*e*)	0.37 (0.23)	0.56 (0.13)	0.58 (0.19)
Stomatal index (*i*)	0.45 (0.19)	< 0.01 (0.33)	0.65 (0.23)
Stomatal area (*s*)	0.24 (0.36)	0.32 (0.22)	0.50 (0.26)

### 3.2 Determination of *g*_max_ Across Arabidopsis Ecotypes by Epidermal Optimization

Across the Arabidopsis ecotypes, *g*_max_ variation was related to epidermal optimization as expected under the hypothesis of adaptive stomatal area fraction (*f*) (Tables S5–S7; Figure 2). Causal and correlative analyses of *g*_max_ with respect to *f* and *s* identified variation in *f* as the major driver of the differences among ecotypes in *g*_max_, and, at the genetic level, the SNPs with greater effect on *g*_max_ on both surfaces were strongly positively correlated with those associated with *f* (*r* = 0.76–0.82; *p* < 0.001). Across ecotypes, higher *g*_max_ and *f* were correlated with higher *d* and higher *s* on one or both surfaces (*r* = 0.24–0.98; *p* < 0.01; Table S6; Figure 2C,D). The *f*‐minimization hypothesis was not supported across ecotypes: *f* values on each surface ranged up to 0.12–0.16, lower than any proposed packing limits [typically 0.33 or 0.5 (Muir et al. 2023)]. Across the ecotypes, the variation in *g*_max_ was independent of the *s*‐*d* trade‐off and orthogonal to that relationship and depended strongly on differences in *f* (*r* = 0.97–0.98 for the abaxial and adaxial surfaces; *p* < 0.001; Table S6; Figure 2A).

### 3.3 Determination of *g*_max_ Variation Across Arabidopsis Ecotypes by Epidermal Development

**FIGURE 3:**
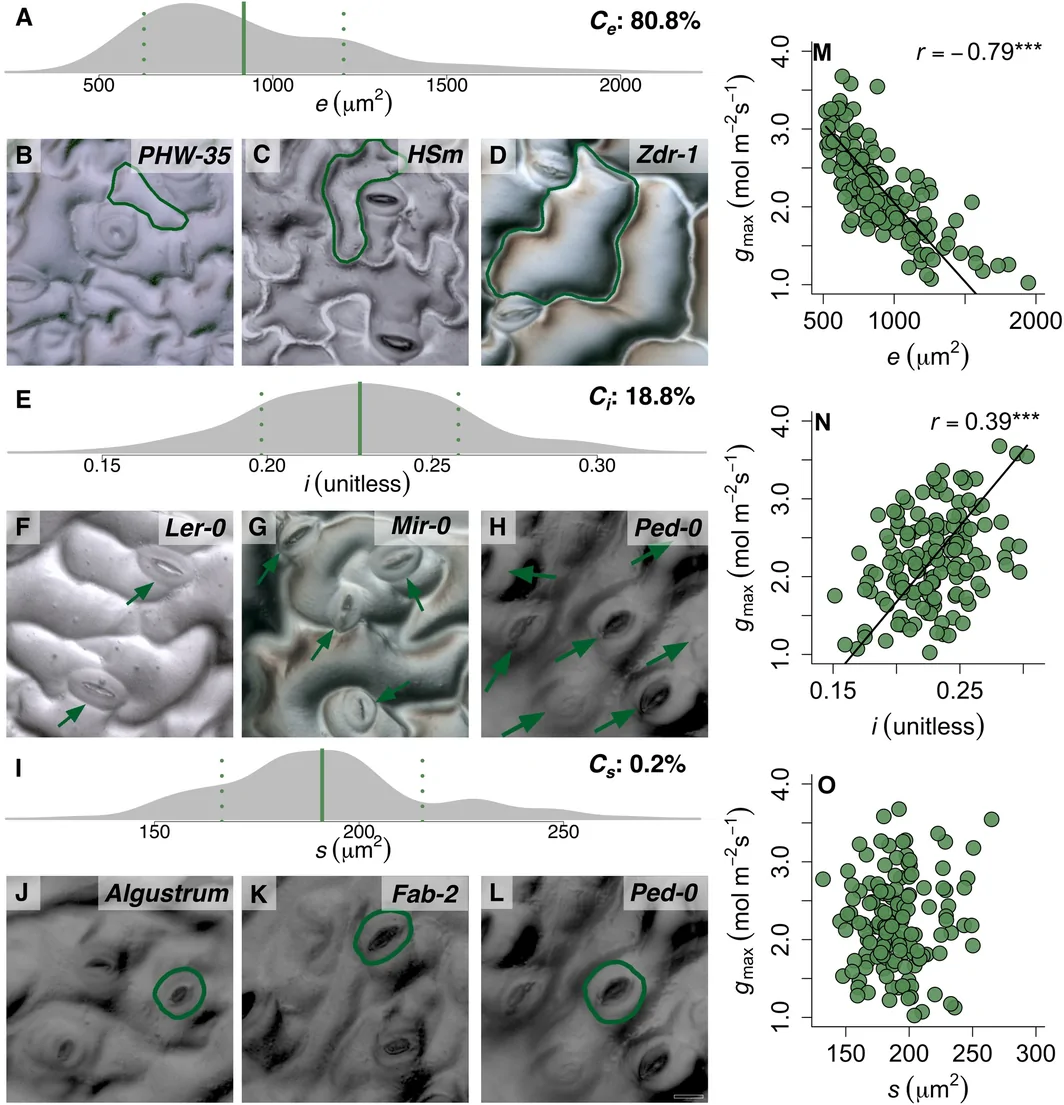
Epidermal development of *g*_max_. Variation in the abaxial anatomical developmental variables contributing to the maximum stomatal conductance, *g*_max_, across 145 ecotypes of *Arabidopsis thaliana* from diverse geographical origins grown under greenhouse conditions. (A) Distribution of epidermal pavement cell sizes, *e* (vertical solid and dotted lines represent the mean ± standard deviation) and (B–D) micrographs of the ecotypes with the lowest, mean and highest values of *e*. (E) Distribution of stomatal index, *i* (vertical solid and dotted lines represent the mean ± standard deviation) and (F–H) micrographs of the ecotypes with the lowest, mean and highest values of *i*. (I) Distribution of stomatal sizes, *s* (vertical solid and dotted lines represent the mean ± standard deviation) and (J–L) micrographs of the ecotypes with the lowest, mean and highest values of *s*. Relationships of *g*_max_ with (M) *e*, (N) *i* and (O) *s*. The causal contribution, *C*, is the median contribution (%) of differences in anatomical variables (*e*, *i* and *s*) to differences in anatomical leaf *g*_max_ in the abaxial surface. Medians were calculated over all possible pairwise comparisons between ecotypes (*n* = 10,440 comparisons). Solid lines represent the standardized major axis fit. ***p < 0.001. The leaf surface micrographs sometimes include distortions due to the 3‐dimensional surface irregularity of the epidermis in Arabidopsis, but this did not interfere with the stomatal measurements. The scale bar on panel L applies to all micrographs (10 μm).

Considering the causes of variation in *g*_max_ across Arabidopsis ecotypes with respect to epidermal development, the hypothesis of multiple developmental drivers was supported (Tables S5 and S6; Figure 3). Across ecotypes, on average, a higher *g*_max_ was driven by smaller *e* and higher *i*, and minimally by differences in *s*. The intrinsic sensitivity analysis of Equation 2 (Figure 1A) shows that all else being equal, the causal determinants of high *g*_max_ would be lower *e*, higher *i* and higher *s* (Table S5). In realized sensitivity analyses, i.e., causal partitioning of the median determination of *g*_max_ given actual variation in all variables, for the abaxial surface, *e* contributed 80.7% and *i* 18.8% to differences in *g*_max_, and *s* contributed negligibly (Table S5; Figure 3A,E,I). For the adaxial surface, *e* contributed 82.4%, *i* 14.8% and *s* still contributed little (4.0%) in determining variation in *g*_max_ (Table S5). Considering total *g*_max_, the strongest contributors were adaxial and abaxial *e* (43.6% and 34.4%, respectively), followed by abaxial and adaxial *i* (10.6% and 8.9%), and < 1% for adaxial and abaxial *s* (Table S5). The observed correlations among stomatal traits were consistent with causal determination of *g*_max_: *g*_max_ was positively correlated with *i* and negatively with *e* for both the abaxial and adaxial surfaces (|*r*| = 0.38–0.85; *p* < 0.001; Table S6; Figure 3M,N), and *g*_max_ was weakly related *s* on the adaxial surface only (*r* = 0.24; *p* < 0.01; Table S6; Figure 3O). We also found correlations between SNP effects on *g*_max_ and developmental traits, supporting the importance of multiple components (Table S7). The SNPs with greatest effects on *g*_max_ for each leaf surface were negatively associated with SNPs influencing *e* (*r* = −0.02 to −0.47; *p* < 0.001), positively associated with SNPs influencing *i* (*r* = 0.04 to 0.22; *p* < 0.001) and weakly positively associated with those influencing *s* (*r* = 0.01 to 0.02; *p* < 0.01) (Table S7).

Stomatal developmental traits were partially inter‐related on each leaf surface, as expected from developmental constraints (Box S1). Thus, *i* and *e* were positively correlated (*r* = 0.19 for the abaxial surface; *p* < 0.05), and *s* and *i* were negatively correlated for the adaxial surface (*r* = −0.20; *p* < 0.05; Table S6).

Developmental trait linkages provide a proximate basis for the weak *s* vs. *d* trade‐off across Arabidopsis ecotypes, observed only on the abaxial surface (Figure 2B). As higher *d* was strongly driven by small *e* and high *i* across ecotypes (|*r*| = 0.39–0.83; *p* < 0.001; Table S6), the observed independence of *s* from *e* and the positive association of *e* and *i* on the abaxial surface would have contributed to a reduced association between *d* and *s* (Table S6; Box Figure S1).

### 3.4 Climatic Adaptation of *g*_max_ Across Arabidopsis Ecotypes: Stress‐Avoidance

**FIGURE 4:**
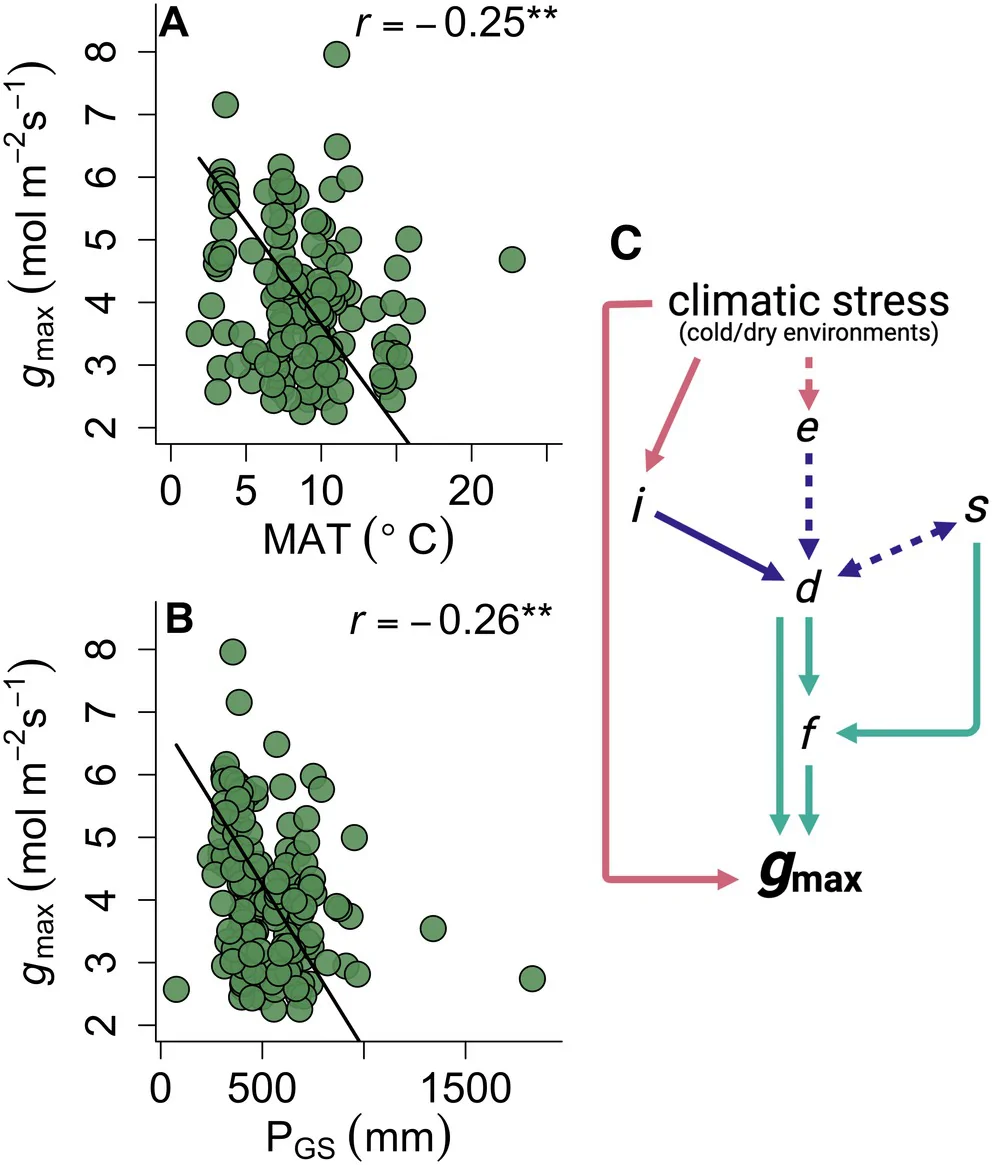
Macroclimate adaptation of the total maximum stomatal conductance, *g*_max_. Relationship of the total *g*_max_ with (A) the mean annual temperature and (B) the precipitation of the growing season, P_GS_, across 145 ecotypes of *Arabidopsis thaliana* from diverse geographical origins grown under greenhouse conditions. Solid lines represent the standardized major axis fit. (C) Summary of the relationships among traits and macroclimate including the *Epidermal Optimization* (Figure 2), *Epidermal Development* (Figure 3), and *Environmental Adaptation* frameworks (Table S6). Solid lines represent positive and dashed lines negative relationships. The macroclimate relationships with mean *i* and *e* are shown in Table S6. **p < 0.01.

Across ecotypes, *g*_max_ and associated traits varied with macroclimate as expected from stress‐avoidance with respect to colder and/or drier locations (Table S6; Figure 4). Across ecotypes, *g*_max_ and *d* were correlated with four of the six tested environmental variables of their native origin (Table S6; Figure 4), i.e., a higher *g*_max_ and/or *d* were related to shorter growing season, colder mean annual and growing season temperatures and lower growing season precipitation (*r* = −0.17 to −0.28; *p* < 0.05 for each leaf surface and/or the total of both surfaces). For the adaxial surface only, a smaller *s* was correlated with larger growing season precipitation and length (*r* = −0.21 and −0.19, respectively; *p* < 0.05) and *e* corresponded to higher mean annual temperatures and longer and wetter growing seasons (*r* = 0.21 to 0.30; *p* < 0.05). The weak *d* vs. *s* trade‐off observed across the Arabidopsis ecotypes could thus not have arisen from coordinated adaptation of high *d* and small *s* to given environmental factors (Table S6; Figure 2B). Notably, larger *f* values for the adaxial surface or averaged for both surfaces corresponded to larger mean annual aridity index, lower mean annual temperature, and shorter, colder and drier growing seasons (*r* = −0.16 to −0.28; *p* < 0.05).

The relationships between stomatal traits and macroclimatic gradients also arose at the genomic level (Table S7), with a correlation of the SNPs with strongest effects on *g*_max_, *d*, *f*, *e* and *i* and those associated with climate variables, including aridity index, growing season precipitation and growing season length (|*r*| = 0.01; *p* < 0.05).

The adaptation of *g*_max_ across ecotypes for stress‐avoidance contrasted with the stress‐resistance observed at the scale of the plant life cycle. Ecotypes from colder and/or drier environments with shorter growing seasons tended to exhibit longer times to flowering at 16°C (FT_16_; *r* = −0.47 and −0.38, respectively; *p* < 0.001; Figure S4; Table S6). Notably, the ecotypes with higher FT_16_ tended to have higher abaxial, adaxial and total *g*_max_ (*r* = 0.58–0.73; *p* < 0.001).

## 4 Discussion

We clarified the adaptation of *g*_max_ and its developmental and mechanistic bases. Refining our understanding of plant stomatal evolution in response to climate change is important for advancement in a wide range of fields, from stress ecology, to crop breeding to ecosystem‐climate modelling (Faralli et al. 2019; Franks et al. 2015; Lang et al. 2024). The integration of frameworks for the developmental and functional bases of stomatal adaptation to macroclimate provides multi‐faceted explanations for variation in *g*_max_ and the stomatal size‐density trade‐off. First, with respect to epidermal optimization, across Arabidopsis ecotypes, a higher *g*_max_ was driven by greater allocation of space to stomata (higher *f*). Developmentally, *g*_max_ was driven especially by small epidermal pavement cells and additionally by a high initiation rate of stomata (high *i*). Ecotypes native to colder and drier climates, which are typically slower growing and require longer times to flowering, possess higher *g*_max_, supporting stress‐avoidance rather than stress‐resistance, i.e., potentially enabling rapid gas exchange, photosynthesis and growth during warmer and wetter periods of the day or of the year (Basu et al. 2016; Xu and Zhou 2008). These results demonstrate the multi‐tiered, dovetailed adaptation of *g*_max_ to climatic stress (Figure 4C). Notably, despite Arabidopsis having its stomatal developmental pathways mapped and mutants characterized, stomatal trait variation across natural ecotypes was not explained by individual genes of large effect, and instead, by polygenic control of stomatal development and *g*_max_ by many loci of small effects, indicated by the relatively high *p*_ve_ for most traits and the strong correlations among stomatal traits at genetic level (Tables 2 and S7) (Atwell et al. 2010; Clo et al. 2019; Solovieff et al. 2013). The polygenic control of natural variation, despite the existence of known mutants with extreme phenotypes, may indicate that single‐gene mutations in loci with high control may be deleterious in natural populations (Legan et al. 2024; Wayne and vonHoldt 2012).

### 4.1 Epidermal Optimization of *g*_max_ Across Arabidopsis Ecotypes

Our tests of hypotheses for the epidermal optimization of *g*_max_ provide a novel perspective relative to the predominant view in the literature. Across ecotypes, a high *g*_max_ on both leaf surfaces was causally driven by high *f* (Tables 1 and S5–S7; Figure 2A,B). We found a *d*‐*s* relationship on the abaxial surface only, as previously reported for a different set of Arabidopsis ecotypes (Dittberner et al. 2018). Thus, in contrast with theory developed for diverse, distantly related species and lineages (de Boer et al. 2016; Franks et al. 2009; Franks and Beerling 2009), i.e., the hypothesis of “minimized *f*” in stomatal evolution, *f* did not approach a packing limit and there was no indication of its minimization or strong constraint. The lack of constraint on *g*_max_ by *f* suggests a potential for evolving yet higher *g*_max_, via greater allocation of the epidermal surface to stomata.

### 4.2 Developmental Determinants of *g*_max_ Variation Across Arabidopsis Ecotypes

We identified both epidermal pavement cell size and stomatal index as drivers of *g*_max_ variation across ecotypes, supporting not only “passive dilution” of stomata by shifts in *e*, but also important shifts in the rate of stomatal precursor cell differentiation (Figure 3). This finding was consistent with a recent study of diverse angiosperm species, implicating these stomatal developmental traits in the evolution of faster growth rates and adaptation to climatic drought (Baird et al. 2024). The independence of *s* from *e*, and the positive association of *e* and *i* on the abaxial surface would have contributed to the limited association between *d* and *s*, i.e., a weak correlation for the abaxial surface only (Figure 2B).

### 4.3 Climatic Adaptation of *g*_max_ Across Arabidopsis Ecotypes

Our study supported trait‐climate adaptation, given the finding in a common garden of correlations in the range of strengths previously reported for key ecophysiological traits, expected to be ecologically meaningful [mean |*r*| within and across studies = 0.26; (cf. Albarrán‐Lara et al. 2015; Rosas et al. 2019)] (Appendix S3). Notably, these correlations occur despite many potential sources of trait‐climate mismatch, and would likely be stronger if microclimate data were available for the native populations (Medeiros et al. 2023).

Our study highlights the contrasting association of stress‐avoidance and stress‐resistance from organ to whole‐plant scale across ecotypes adapted to contrasting climates. While *Arabidopsis thaliana* as a species is considered relatively “stress‐avoidant” (Bouzid et al. 2019), across Arabidopsis ecotypes, those adapted to cold and dry macroclimates tend to show greater stress‐resistance at whole‐plant scale, with respect to their slow growth and long time to flowering (Exposito‐Alonso 2020; Kazan and Lyons 2016; Sanda et al. 1997; Sartori et al. 2019), and indeed, certain ecotypes require vernalization to trigger flowering (i.e., prolonged exposure to cold, or overwintering) (Capovilla et al. 2015; Song et al. 2012). Yet, such cold‐ and dry‐adapted ecotypes possessed high *g*_max_, which would enable greater stress‐avoidance at leaf scale (Table S6; Figure 4A,B), which would be beneficial despite these ecotypes having a longer vegetative growth period because a lower portion of that period would be climatically favourable (Song et al. 2012). Indeed, our findings for higher *g*_max_ as an adaptation to colder and drier climates is consistent with studies of other plant systems, e.g., higher maximum rates of photosynthesis in semi‐desert shrubs relative to those in moist climates (Grubb 1998), but differs from others, e.g., Hawaiian forest trees and shrubs, among which species of dry forests had lower *g*_max_ than those in wet forests, consistent with chronic low resource availability in the dry forest, unrelieved by diurnal and/or seasonal moist periods, such that avoidance strategies would provide few benefits (Medeiros et al. 2019). Notably, the combination of stress‐avoidance at organ scale and stress‐resistance at whole plant scale has been previously established in other contexts: thus, the stress‐resistant cactus flushes roots when water is available and sheds them during dry periods, a root‐level avoidance behaviour; and California *Ceanothus*, one of the most drought‐tolerant species of its ecosystem, shows sensitive stomatal closure when water is not available and high conductance when water is available, i.e., leaf‐level avoidance (Zailaa et al. 2025). Among Arabidopsis ecotypes, low leaf area allocation may confer whole plant resistance to stressful climates, corresponding to stronger root allocation and high leaf mass per area, and a higher *g*_max_ would contribute to faster leaf gas exchange that would mitigate the associated reduction of relative growth rate (Fletcher et al. 2022).

### 4.4 Testing Multiple Frameworks for Explaining the Trade‐Off Between Stomatal Size and Density

Our study tested multiple hypotheses to explain the *s*‐*d* trade‐off, established across many, but not all, plant systems (Table S1). Across Arabidopsis ecotypes, we found a negative *s*‐*d* association only for the abaxial leaf surface. This pattern can be explained under the epidermal developmental framework by the weakness of relationships between *e* and *s* and between *i* and *s*, and the positive relationship between *e* and *i* (Box Figure S1). We conclude that relationships between developmental traits are thus context‐specific, and amenable to selection under different pressures in different lineages (cf. Schwenk and Wagner 2004). The other frameworks for the *s*‐*d* association did not provide explanations for the observed pattern among Arabidopsis ecotypes. The optimization of the epidermis among Arabidopsis ecotypes involves increasing *f* as a driver of higher *g*_max_ and does not rely on, or force an *s*‐*d* trade‐off, as would be expected if *f* were minimized (de Boer et al. 2016; Franks and Beerling 2009). Further, adaptation to macroclimate did not drive an *s*‐*d* trade‐off: a higher *d* was associated with colder and drier macroclimate, consistent with stress‐avoidance and smaller *s* with less stressful macroclimates, only for the adaxial surface. Studies within and across species have indicated that a smaller *s* may contribute to stress tolerance via more rapid responses and potentially higher water use efficiency under changing irradiance (Drake et al. 2013; Lawson and Blatt 2014)—yet such adaptation may not be important for Arabidopsis in typically open habitats (Castilla et al. 2020; Kang et al. 2023).

### 4.5 Limitations of the Study and Future Directions

Our resolution of trait‐climate associations in a common garden supports adaptation, in the sense of environmentally‐aligned heritable phenotypic variation, and additional approaches would be needed to support adaptation in a narrower sense, e.g., the demonstration that stomatal traits provide a home site advantage to given ecotypes (Appendix S3). Further work is needed to generalize across other herbaceous or crop species and to enable comparisons with other life forms such as trees (e.g., Meng et al. 2025). The dovetailing frameworks of function, development and genetic architecture can thus be applied and extended in a broad range of future studies.

## 5 Conclusion

Stomata are a model system exemplifying and clarifying how ecologically‐important traits scale up to higher level functions and adapt to climate. Across Arabidopsis ecotypes, a high *g*_max_ would confer stress‐avoidance under cold and/or dry climates. We resolved low *e* and high *i* as leaf developmental variables with major roles in determining high epidermal allocation to stomata and high *g*_max_. Our findings suggest a pleiotropic genetic basis for *g*_max_ across Arabidopsis ecotypes. Across Arabidopsis ecotypes, dovetailing drivers of *g*_max_ showed the integration of genetics, development, function and climate adaptation, providing lessons for stomatal adaptation, rendering important complexity more tractable and pointing to new avenues for clarifying the mechanisms and implications of variation in traits that contribute importantly to ecological adaptation.

## Author Contributions

Conceptualization: C.D.M., T.N.B., C.V., L.S. Formal analysis: C.D.M., T.N.B., F.V. Investigation: C.D.M., K.S., L.R.F. Visualization: C.D.M., T.N.B., F.V. Funding acquisition: C.V., D.V., F.V., M.P., E.B., E.K., L.S. Project administration: C.V., D.V., F.V., M.P., E.B., E.K., L.S. Supervision: C.V., D.V., F.V., E.B., E.K., L.S. Writing – original draft: C.D.M., T.N.B., L.S. Writing – review and editing: C.D.M., T.N.B., K.S., C.V., D.V., F.V., L.R.F., L.S.

## Funding

This work was supported by the National Institute of Food and Agriculture, 2020‐67013‐30913, Hatch Project number 1016439. Ciência sem Fronteiras, 202813/2014‐2. Directorate for Biological Sciences, 1457279, 1557906, 1951244, 2017949, 2307341.

## Conflicts of Interest

The authors declare no conflicts of interest.

## Supporting information


**Appendix S1:** ele70453‐sup‐0001‐Appendix S1.docx.

**Appendix S2:** ele70453‐sup‐0002‐Appendix S2.xlsx.

**Appendix S3:** ele70453‐sup‐0003‐Appendix S3.docx.

## Data Availability

All trait and climate data collected for this paper are available from the Dryad Digital Repository: https://doi.org/10.5061/dryad.kprr4xhfb (Medeiros et al. 2026a) and the relevant code is available on Zenodo: https://doi.org/10.5281/zenodo.17288996 (Medeiros et al. 2026b).
