# Supplementary material for: Dovetailing the Genetic, Developmental and Functional Bases of the Adaptation of Stomatal Traits to Climate
Source: Ecol Lett. 2026 Sep 16;29(9):e70453. doi: 10.1111/ele.70453 (PMC13579612; doi:10.1111/ele.70453)
Supplement: Supplementary file 1 [file ELE-29-0-s001.docx]

***Ecology Letters***

**Appendix S1**

**Supplementary Information for:**

**Dovetailing the genetic, developmental and functional bases**

**of the adaptation of stomatal traits to climate**

**Authors:** Camila D. Medeiros^1,2*^, Thomas N. Buckley^3^, Kevin Sartori^4^, Cyrille Violle^4^, Denis Vile^5^, François Vasseur^4,5^, Leila R. Fletcher^6^, Matteo Pellegrini^7^, Etienne Baron^4^, Elena Kazakou^4^, Lawren Sack^1*^

**Supplementary Methods, Supplementary Box S1, Tables S1-S2 and Figures S1-S4**

**Supplementary Methods**

*Plant growing conditions*

We grew 145 ecotypes of *Arabidopsis thaliana* from the 1,001 Genomes Consortium populations (Alonso-Blanco *et al.* 2016) selected to represent the extent of the natural distribution of the species (Fig. 1D), and the RegMap populations (Horton *et al.* 2012) in a common greenhouse. Seeds were sown in moist organic compost and stratified in a cold chamber at 4°C for four days. Four seedlings per genotype were then transferred to individual pots filled with organic compost (Neuhaus N2) and watered twice a week for the duration of the experiment (140 days from sowing to harvest). Pots were distributed across four greenhouse tables in blocks, with one replicate per ecotype per table. The greenhouse temperature was controlled at 18°C during the day and 16°C during the night, and tables were rotated daily to reduce block effects within the greenhouse. A supplemental lighting system was used to maintain a constant 12.5 h day-length. For trait measurements, we use three of the four individuals grown in the greenhouse.

*Image analysis*

The leaves were fixed in formalin–acetic acid–alcohol solution (FAA) for at least two days and then transferred to a pure glycerol medium for storage. All traits were measured manually using the software ImageJ (<http://imagej.nih.gov/ij/>). Stomatal dimensions were obtained from four stomata per image. Each image was divided into four quadrants and one stoma per quadrant was measured to reduce selection bias. To represent the large variation in epidermal pavement cell shape and size in Arabidopsis (Pillitteri & Dong 2013), for each image we counted the number of epidermal pavement cells and calculated an average epidermal cell area by dividing the area of the image not occupied by stomata by the number of epidermal pavement cells. From those measurements, we calculated the stomatal index (*i*, also known as “stomatal initiation rate”) as the number of stomata divided by the sum of epidermal pavement cells and stomata (Sack & Buckley 2016). Because the images were not always clear enough to count all of the epidermal cells, we sometimes measured less than three replicates per ecotype (see detailed sample sized per ecotype on the Ecotype dataset available on Dryad), but for more than 82% of the ecotypes, we were able to measure three individuals. We calculated the stomatal area fraction (*f*) as the product of the stomatal area and density, and the maximum theoretical stomatal conductance (*g*_max_) as:

$$\frac{bmds}{s^{0.5}} Eqn 4$$

where *b* ≡ *D*/*ν*, *D* (2.55⋅10^-5^ m^2^ s^-1^) is the binary diffusivity of water vapor in air, *ν* (2.45⋅10^-2^ m^3^ mol^-1^) is the molar volume of air, and *m* is a dimensionless scaling factor representing conserved allometries among guard cell and stomatal pore dimensions (*m* ≈ 0.43 for non-grasses, which have kidney-bean shaped guard cells, and ≈ 1.22 for grasses, with their dumb-bell shaped guard cells) (Franks *et al.* 2009a; Franks & Beerling 2009; Franks & Farquhar 2007; McElwain *et al.* 2016; Sack & Buckley 2016; Taylor *et al.* 2012).

*Extraction of macroclimate variables*

We obtained ecotype collection coordinates from the 1,001 Genomes Consortium (Alonso-Blanco *et al.* 2016) and RegMap panel (Horton *et al.* 2012) and used R software [version 3.4.4 (R Core Team 2020)] to extract five salient macroclimate variables considered in previous studies of Arabidopsis for considering the linkage of traits with cold and/or dry climate ecotypes (e.g., Dittberner *et al.* 2018; Fletcher *et al.* 2022; Lasky *et al.* 2012; Sartori *et al.* 2019): mean annual aridity index, annual and growing season mean temperature and precipitation and the length of the growing season (Hijmans *et al.* 2005; Trabucco & Zomer 2018). The raster layers with the same resolution were stacked using the *stack* function and the environmental parameters for each geographic coordinate were extracted using the *extract* function from the ‘*raster’* package [version 3.6.23;(Hijmans & van Etten 2012)]. Additionally, the growing season was considered as the months that have abundant soil moisture (mean precipitation in mm ≥ 2 × mean temperature in degrees Celsius) and minimum temperature above 4ºC, as below that temperature water becomes too viscous to pass through membranes (Lasky *et al.* 2012).

*Statistical and comparative analyses*

Using the kinship matrix in generalized mixed-effects regression analyses is more powerful in reducing types I and II errors in datasets with complex familial relationships and population structure of relatedness than other frameworks, such as phylogenetic generalized least-squares analyses (Yu *et al.* 2006).

To quantify the trait variation across ecotypes we performed ANOVAs using the *Anova* function from the ‘car’ package (version 3.1.0), with functional traits coded as the dependent variable and ecotype as the independent variable, followed by a Tukey test at 5% probability using functions available in the ‘multcomp’ package [version 1.4.20; (Hothorn *et al.* 2008; Sokal & Rohlf 2012)]. To test for differences in traits between the abaxial and adaxial surfaces, we performed paired *t*-tests using the *t.test* function from the ‘stats’ package [version 4.2.1; (Sokal & Rohlf 2012)]. We also calculated parametric and non-parametric coefficients of variation of anatomical traits across ecotypes (respectively, CV = $\frac{standard deviation}{mean} \times100$ and NPCV = $\frac{median absolute deviation}{|mean|} \times100$), using the *npcv* function in the ‘MSnbase’ package [version 2.24.2; (Gatto & Lilley 2012)]. To test for differences in CVs between the abaxial and adaxial surfaces, we performed two tests using the ‘cvequality’ package [version 0.2.0; (Marwick & Krishnamoorthy 2019)], an asymptotic test for the equality of coefficients of variation from *k* populations (Feltz & Miller 1996) and a modified signed-likelihood ratio test (Krishnamoorthy & Lee 2014).

*Causal partitioning analysis*

To determine the realized sensitivity of *g*_max_ to leaf anatomical parameters *e*, *i* and *s*, and of *g*_max_ to *f* and *s*, we conducted causal partitioning analyses of the variation in *g*_max_ contributed by the underlying variables (Buckley & Diaz-Espejo 2015) (Table S5). In this approach, a finite difference *δy* in some function *F* of *N* underlying variables *x*_j_ (*j*=1 to *N*) between two states—an initial or “reference” state (*y*_r_ = *F*(*x*_1r_, *x*_2r_, ...)) and a final or “comparison” state (*y*_c_ = *F*(*x*_1c_, *x*_2c_, ...))—is partitioned into contributions from changes in each *x* variable, by numerically integrating the partial derivatives of *F* with respect to each *x* variable (∂*F*/∂*x*_j_) over an imaginary path between the two states, with each variable assumed to change linearly between its values in each state. Each contribution is expressed relative to the sum of all contributions, so the contributions add up to 100%. Thus, the % contribution of variable *x*_k_ (*C*[*x*_k_]) to the difference in *y* (*δy* = *y*_c_ – *y*_r_) between the two states is given by:

$$C\left( x_{k} \right)=100\cdot\frac{\int_{r}^{c} \frac{\partial F}{\partial x_{k}}dx_{k}}{\sum_{j=1}^{N} \int_{r}^{c} \frac{\partial F}{\partial x_{j}}dx_{j}}=100\cdot\frac{\int_{r}^{c} \frac{\partial F}{\partial x_{k}}dx_{k}}{y_{c}-y_{r}} Eqn 5$$

We used this method to partition: (i) abaxial (and adaxial) *g*_max_ into contributions from abaxial (and adaxial) values of *e*, *i*, and *s*, and of *f* and *s*, (ii) total *g*_max_ into contributions from abaxial and adaxial *g*_max_, and (iii) total *g*_max_ into contributions from both abaxial and adaxial values of *e*, *i* and *s*, and of *f* and *s*. In each case, we repeated these calculations for every possible pairwise comparison between ecotypes (for 145 ecotypes, this gives 145!/[143!×2!] = 145×144/2 = 10,440 pairwise comparisons), with values for the two ecotypes in each pair representing the reference and comparison states respectively, and each numerical integration using 1000 steps. We then calculated the median contributions (values of *C*) for each variable over the 10,440 comparisons. A negative *C* for an underlying variable *x*_j_ indicates that *y* usually changed in the direction opposite to that predicted by the signs of ∂*F*/∂*x*_j_ and of the change in *x*_j_. Although the contributions calculated for a comparison between two given ecotypes always sum to 100%, the median values of those contributions across all such comparisons may not sum exactly to 100%. A negative *C* for an underlying variable indicates that *g*_max_ differences between ecotypes tended to be opposite in direction to that expected by the differences in that variable; thus, the effect of that variable was “contracausal”, and its influence overshadowed by shifts in other underlying variables.

*Intrinsic sensitivity analysis*

To quantify the intrinsic sensitivity of *g*_max_ to its determinants, we computed sensitivity coefficients (relativized derivatives). For Equation 1, the expressions are:

$$\frac{\partial\ln g_{\text{max}}}{\partial\ln f}=1$$

$$\frac{\partial\ln g_{\text{max}}}{\partial\ln s}=-\frac{1}{2}$$

For Equation 2, the expressions are:

$$\frac{\partial\ln g_{\text{max}}}{\partial\ln d}=1$$

$$\frac{\partial\ln g_{\text{max}}}{\partial\ln s}=\frac{1}{2}$$

*Genetic analyses*

For genetic analyses we used a single nucleotide polymorphism (SNP) dataset produced by merging the 1,001 Genomes dataset [whole-genome sequencing, around 12 million SNPs in total (Alonso-Blanco *et al.* 2016)] and the RegMap dataset [around 250 thousand SNPs (Horton *et al.* 2012)]. We used PLINK [http://pngu.mgh.harvard.edu/purcell/plink/ (Purcell *et al.* 2007)] to filter SNPs with minor allele frequency > 5%, resulting in a total of 1,709,387 SNPs. We used GEMMA [genome-wide efficient mixed model association; https://github.com/genetics-statistics/GEMMA (Zhou & Stephens 2012)] to perform classical GWAS (linear mixed-model, LMM) analyses to test associations of SNPs with anatomical traits (*g*_max_, *d*, *f*, *e*, *i* and *s*) and key macroclimate variables of ecotype origins. GEMMA was also used to perform polygenic GWAS (Bayesian sparse linear mixed-model, BSLMM) to estimate SNP effects on each variable (Zhou *et al.* 2013). We used the top 1% SNPs (0.5% strongest negative and 0.5% strongest positive) to extract a list of genes in which these SNPs are located and find the stomatal traits with weak and/or high statistical enrichment of genes related to molecular functions and biological processes (Table S2). SNPs with a *p*-value below the threshold established after a Bonferroni correction [false discovery rate (FDR) < 0.05] were considered significantly related to the phenotype. Manhattan plots were created using the ‘*manhattan*’ function in the *qqman* package (Turner 2018). Narrow-sense heritabilities were estimated by chip heritabilities from the BSLMM analyses (Zhou *et al.* 2013).­_­_

All GWAS results were analyzed and plotted using R. Gene Ontology (GO) analyses were performed using GOrilla [http://cbl-gorilla.cs.technion.ac.il/ (Eden *et al.* 2007, 2009)].

**Box S1.** Developmental constraints resulting in strong associations among epidermal pavement cell area, *e*, stomatal index, *i*, and stomatal area, *s*

We hypothesized relationships that may arise across ecotypes between *e*, *i* and *s* due to developmental trait correlations. These relationships would in turn influence the association of *s* and *d*. First, we hypothesized a positive *e* and *s* association, based on studies across species that showed that cell sizes are coordinated, potentially due to association with genome size (Beaulieu *et al.* 2008). Given the importance of *e* as a negative driver of *d*, a positive *e*-*s* relationship may thus contribute to a negative relationship of *d* with *s* (Box Fig. S1A; Table 1). Second, we hypothesized a negative *i* and *s* association given that a high *i* may be generated by meristemoid lineages with more iterations of division, resulting in smaller stomata (Bergmann & Sack 2007). Given the importance of *i* as a positive driver of *d*, a negative *i*-*s* relationship may thus contribute to a negative relationship of *d* with *s* (Box Fig. S1B; Table 1). Finally, we hypothesized a positive *i* and *e* association, as a higher *e* would arise from a greater duration of pavement cell expansion after cell differentiation ceased (Carins Murphy *et al.* 2016, 2017), enabling in that time the differentiation of greater numbers of stomata from meristemoid epidermal cells (Bergmann & Sack 2007; Lau & Bergmann 2012; Nadeau 2002). Given the contrary influences on *d* of *i* and *e* (positive and negative, respectively) their positive relationship would destabilize an *s*-*d* association (Box Fig. S1C; Table 1).

**Box Fig. S1.** Relationships among the anatomical developmental variables that determine stomatal density (*d*) and maximum stomatal conductance (*g*_max_), and that would contribute to a negative association between stomatal size and density by Eqn 2, and results for Arabidopsis ecotypes that explain why *s* and *d* are partially decoupled across ecotypes, with a weak trend only on the abaxial surface, and not on the adaxial surface or considering both surfaces combined (Fig. 2D). In principle, because a low *e* and a high *i* drive an increase in *d*, (**A**) a positive *e*-*s* relationship due to cell size coordination or (**B**) a negative *i*-*s* relationship due to smaller stomata arising from more iterative divisions would result in a negative *s*-*d* association, whereas, because *i* and *e* have converse influences on *d*, (**C**) a positive *i*-*e* relationship would not drive any association of *s* and *d*. Thus, the lack of associations for abaxial and adaxial surfaces for 145 ecotypes of Arabidopsis of (**D, G**) *e* and *s* and (**E, H**) *i* and *s*, the association of (**F, I**) *i* and *e*, would contribute to decoupling of *s* and *d* across ecotypes. Solid lines represent the standardized major axis fit. **p* < 0.05.

**Table S1.** Published studies showing the association of stomatal density, *d*, and area, *s*, within and across species. When *s* is not presented in the paper, we report guard cell length (GC_L_), guard cell width (GC_W_) or stomatal pore length (SP_L_) as proxies; when the maximum stomatal conductance, *g*_max_, is not presented, we report the stomatal pore index (SPI) as proxy. For each study, we report whether the study found evidence of an *s*-*d* trade‐off and provide the comparative context, statistical test used, and references.­

| **Study** | **Comparative context** | **Statistical test** | **Stomatal traits measured** | ***s*-*d* trade-off?** |
| --- | --- | --- | --- | --- |
| (Grubb *et al.* 1975) | 60 species of Eudicot evergreen trees and shrubs growing in a Japanese warm temperate rainforest | Correlation test (OLS regression) | *d* and GC_L_ | Yes  *r* = -0.55; *p* < 0.001 |
| (Muchow & Sinclair 1989) | 10 *Sorghum bicolor* varieties grown in the field in Gainesville, Florida-USA | Pearson or Spearman correlation tests (OLS regression) | *g*_max_, *d* and *s* | Yes  *r* = -0.78; *p* < 0.01 |
| (Hetherington & Woodward 2003) | Data from several papers and personal observations.  Includes a diverse set of species: Amphistomatous, hypostomatous, grasses and fossil species | Not reported | *d* and GC_L_ | Yes  *r* = -0.71; *p* not reported |
| (Sack *et al.* 2003) | 10 diverse woody and vine species sampled from Harvard Forest, Massachussets-USA | Pearson or Spearman correlation tests (OLS regression) | *g*_max_, *d* and *s* | Yes  *r* = -0.87; *p* < 0.01 |
| (Ohsumi *et al.* 2007) | 67 genotypes of *Zea mays* grown in a plantation in Japan | Correlation tests (OLS regression) | *g*_s_, *d* and GC_L_ | Yes  *r* = -0.59; *p* < 0.001 |
| (Dunbar‐Co *et al.* 2009) | Seven endemic Hawaiian Plantago species, 6 grown under controlled conditions in a greenhouse and 1 sampled from the field | Correlations tests not performed, so we analyzed the published data using  Pearson correlation tests (OLS regression; log-transformed) | *d*, GC_L_ and SP_L_ | No  *r* = -0.74; *p* = 0.06 |
| (Franks *et al.* 2009b) | Individuals of *Eucalyptus globulus* grown at two plantations in two field sites (in low and in high rainfall) in Australia | Correlation tests | *g*_max_, *d*, *s* and GC_L_ | Yes for both high and low rainfall  *r* ranged from -0.74 to -0.88; *p* < 0.001 |
|  |  |  |  |  |
| (Camargo & Marenco 2011) | 35 woody species from a tropical forest site in the Brazilian Amazon | Correlation tests (OLS regression) | *d* and GC_L_ | Yes *r* = -0.60; *p* < 0.05 |
| (Dow *et al.* 2014) | 9 genotypes of *Arabidopsis thaliana* grown in a growth chamber in Stanford, California-USA | Pearson or Spearman correlation tests (OLS regression) | *g*_max_, *d* and *s* | No  *r* = -0.31; *p* > 0.05 |
| (Wang *et al.* 2015) | 870 species across a latitudinal gradient in China | Pearson correlation tests (OLS regression) | *d* and GC_L_ | Yes  *r* = -0.71; *p* <0.001 |
| (McElwain *et al.* 2016) | 19 gymno- and angiosperm species, 9 grown in pots in a production glasshouse at the UCD Rosemount Environmental Research Station, Ireland, and 10 grown at  the National Botanic Gardens Glasnevin, Ireland | Correlations tests not performed, so we analyzed the published data using  Pearson correlation tests (OLS regression; log-transformed) | *g*_max_, *d* and SP_L_ | Yes  *r* = -0.69; *p* < 0.01 |
| (McKown *et al.* 2016) | 9 *Scaevola* species sampled in their natural, native environments on the islands of Hawai‘i, Kaua‘i, Maui, and O‘ahu | Correlations tests not performed, so we analyzed the published data using  Pearson correlation tests (OLS regression) | SPI, *d* and SP_L_ | No  *r*_aba_ = -0.55; *p* = 0.12  *r*_ada_ = 0.23; *p* = 0.63 |
| (Dittberner *et al.* 2018) | 330 genotypes of Arabidopsis grown in a growth chamber | Pearson correlation tests (OLS regression) | *d* and *s* | Yes  *r* = -0.50; *p* < 0.001 |
| (Kardiman & Ræbild 2018) | Seedlings of 11 rainforest species grown in the nursery of Harapan Rainforest, Sumatra, in a tropical lowland rainforest | Pearson correlation tests (OLS regression); tests of *g*_s-op_-*d* and *g*_s-op_-*s* were not performed, so we analyzed the published data using  Pearson correlation tests (OLS regression) | *g*_s-op_, *d*, *s*, GC_L_ and GC_w_ | Yes  *r* = -0.73; *p* = 0.017  (one species was removed from this analysis, so we did the same for the analyses we performed using their published data) |
| (Liu *et al.* 2018) | 737 plant species from nine plant communities, from tropical to cold temperate forests along a 3700 km transect in China | Correlations tests not performed, so we analyzed the published data using Pearson correlation tests (OLS regression) | *d*, *s* and *f* | Yes  *r* = -0.33; *p* < 0.001 |
| (Carignato *et al.* 2019) | 8 *Eucalyptus* varieties of potted plants in the field in Huelva, Spain | Pearson or Spearman correlation tests (OLS regression) | *g*_max_, *d* and *s* | No  *r* = -0.52; *p* > 0.05 |
| (Henry *et al.* 2019) | 15 ecologically and morphologically diverse woody species grown in the UCLA Plant Growth Center greenhouse in Los Angeles, CA-USA | Pearson or Spearman correlation tests (OLS regression) | *g*_max_, *d* and *s* | No  *r* = -0.48; *p* > 0.05 |
| (Medeiros *et al.* 2019) | 20 native species from a wet montane forest and 15 from a lowland dry forest in Hawaii, including 3 species of tree ferns | Pearson correlation tests (OLS regression) | *g*_max_, *d*, *e*, *i*, *s*, GC_L_, GC_w_, SP_L_ | No  *r*_all_ = -0.05; *p*_all_ > 0.05  *r*_wet_ = -0.16; *p*_wet_ > 0.05  *r*_dry_ = -0.16; *p*_dry_ > 0.05 |
| (Murray *et al.* 2020) | 75 diverse woody species sampled from the field across four biomes in Fiji, Puerto Rico, Alaska and Oregon-USA | Pearson or Spearman correlation tests (OLS regression) | *g*_max_, *d* and *s* | Yes  *r* = -0.84; *p* < 0.001 |
| (Yin *et al.* 2020) | 45 woody species from a forest site in the middle of the Loess Plateau in China | Pearson correlation tests (OLS regression) and phylogenetic independent contrasts (PICs) | *g*_max_, *d* and GC_L_ | Yes  *r*_ols_ = -0.59; *p*_ols_ < 0.0001 *r*_pic_ = -0.44; *p*_pic_ < 0.01 |
| (Machado *et al.* 2021) | 30 tree species sampled from a fragment of Cerrado in Rio Verde, Goiás-Brazil | Pearson or Spearman correlation tests (OLS regression) | *g*_max_, *d* and *s* | Yes  *r* = -0.79; *p* < 0.001 |
| (Ochoa *et al.* 2024) | 15 *Quercus* species grown in a common garden in Claremont, California-USA | Pearson or Spearman correlation tests (OLS regression) | *g*_max_, *d* and *s* | No  *r* = -0.09; *p* > 0.05 |

**Table S2.** Summary of the gene ontology (GO) analysis results for the phenotypic traits that had a significant enrichment.

| **Trait/ GO Root** | **GO Category** | **GO group ID** | ***p*-value** |
| --- | --- | --- | --- |
| ***Total maximum theoretical anatomical stomatal conductance (g*_max total_*)*** | | | |
| Biological process | Cellular process involved in reproduction in multicellular organism | GO:0022412 | 4.91E-04 |
| Biological process | Cell cycle process | GO:0022402 | 1.99E-04 |
| Biological process | Meiotic cell cycle process | GO:1903046 | 8.34E-05 |
| Biological process | Meiosis II cell cycle process | GO:0061983 | 4.91E-04 |
| Biological process | Meiotic nuclear division | GO:0140013 | 4.91E-04 |
| Biological process | Male meiotic nuclear division | GO:0007140 | 4.91E-04 |
| Biological process | Meiosis II | GO:0007135 | 4.91E-04 |
| Biological process | Male meiosis II | GO:0007142 | 4.91E-04 |
| Biological process | Organelle fission | GO:0048285 | 4.91E-04 |
| Biological process | Nuclear division | GO:0000280 | 4.91E-04 |
| ***Stomatal density in the adaxial surface (d*_ada_*)*** | | | |
| Biological process | RNA splicing, via transesterification reactions | GO:0000375 | 4.58E-04 |
| Biological process | RNA splicing, via transesterification reactions with bulged adenosine as nucleophile | GO:0000377 | 4.58E-04 |
| Biological process | mRNA splicing, via spliceosome | GO:0045292 | 2.57E-04 |
| ***Epidermal pavement cell area in the abaxial surface (e*_aba_*)*** | | | |
| Molecular function | Transferase activity | GO:0016740 | 7.86E-04 |
| Biological process | Cellular process | GO:0009987 | 9.11E-05 |
| Biological process | Regulation of metabolic process | GO:0019222 | 9.18E-04 |
| ***Epidermal pavement cell area in the adaxial surface (e*_ada_*)*** | | | |
| Cellular component | Cell part | GO:0044464 | 1.72E-06 |
| Cellular component | Intracellular part | GO:0044424 | 3.27E-05 |
| Cellular component | RNA polymerase III transcription factor complex | GO:0090576 | 3.77E-04 |
| Cellular component | Transcription factor TFIIIC complex | GO:0000127 | 5.89E-05 |
| Cellular component | Nucleus | GO:0005634 | 3.07E-05 |
| Molecular function | Catalytic activity | GO:0003824 | 3.63E-05 |
| Molecular function | Transferase activity | GO:0016740 | 2.05E-04 |
| Biological process | Cellular process | GO:0009987 | 7.37E-06 |
| ***Mean epidermal pavement cell area (e*_mean_*)*** | | | |
| Cellular component | Nucleus | GO:0005634 | 1.98E-04 |
| Cellular component | Extrinsic component of endoplasmic reticulum membrane | GO:0042406 | 9.61E-04 |
| Molecular function | Single-stranded DNA 5’-3’ exodeoxyribonuclease activity | GO:0045145 | 9.61E-04 |
| Molecular function | Thiaminase activity | GO:0050334 | 9.61E-04 |
| Biological process | Ceramide catabolic process | GO:0046514 | 9.47E-04 |
| ***Stomatal index in the abaxial surface (i*_aba_*)*** | | | |
| Cellular component | Golgi apparatus | GO:0005794 | 5.41E-04 |
| Biological process | Regulation of fatty acid biosynthetic process | GO:0042304 | 5.33E-05 |
| Biological process | Brassinosteroid homeostasis | GO:0010268 | 1.81E-04 |
| Biological process | Regulation of fatty acid metabolic process | GO:0019217 | 4.48E-04 |
| ***Stomatal index in the adaxial surface (i*_ada_*)*** | | | |
| Cellular component | Root hair | GO:0035618 | 9.68E-04 |
| Molecular function | Catalytic activity | GO:0003824 | 5.32E-06 |
| Molecular function | Hydrolase activity | GO:0016787 | 2.11E-05 |
| Molecular function | Glucosidase activity | GO:0015926 | 8.22E-04 |
| Biological process | Peptidyl-serine phosphorylation | GO:0018105 | 4.11E-04 |
| Biological process | Peptidyl-serine modification | GO:0018209 | 4.89E-04 |
| Biological process | Response to lead iron | GO:0010288 | 9.75E-04 |
| ***Mean stomatal index (i*_mean_*)*** | | | |
| Cellular component | Intracellular part | GO:0044424 | 1.22E-04 |
| Cellular component | Intracellular organelle part | GO:0044446 | 1.42E-04 |
| Cellular component | Organelle part | GO:0044422 | 1.50E-04 |
| Cellular component | Golgi apparatus | GO:0005794 | 3.31E-04 |
| Cellular component | Endosomal part | GO:0044440 | 4.28E-04 |
| Cellular component | Cell part | GO:0044464 | 4.62E-04 |
| Biological process | Regulation of chromosome organization | GO:0033044 | 9.65E-05 |
| Biological process | Cellular process | GO:0009987 | 1.85E-04 |
| Biological process | Regulation of organelle organization | GO:0033043 | 2.78E-04 |
| Biological process | Signal transduction | GO:0007165 | 5.80E-04 |
| Biological process | Glutamine family amino acid metabolic process | GO:0009064 | 8.25E-04 |
| ***Stomatal area in the adaxial surface (s*_ada_*)*** | | | |
| Molecular function | Phosphotransferase activity, alcohol group as acceptor | GO:0016773 | 9.06E-04 |
| ***Mean stomatal area (s*_mean_*)*** | | | |
| Molecular function | Oxidoreductase activity, acting on paired donors, with incorporation of reduction of molecular oxygen | GO:0016705 | 9.37E-04 |
| Molecular function | Oxidoreductase activity, acting on paired donors, with incorporation of reduction of molecular oxygen, 2-oxoglutamate as one donor, and incorporation of one atom each of oxygen into both donors | GO:0016706 | 9.37E-04 |

**Figure S1.**

Variation in anatomical variables measured on the abaxial and adaxial surfaces of 145 ecotypes of *Arabidopsis thaliana* from diverse geographical origins grown under greenhouse conditions. (**A**) Maximum theoretical anatomical stomatal conductance, *g*_max_, (**B**) stomatal area fraction, *f*, (**C**) stomatal density, *d*, (**D**) epidermal pavement cell size, *e*, (**E**) stomatal initiation rate, *i*, and (**F**) stomatal size. Each point represents one ecotype (paired *t*-test; table S4). **p* < 0.05; ***p* < 0.01; *** *p* < 0.001.

**Figure S2.**

Genome wide association mapping of variation in abaxial anatomical maximum stomatal conductance, *g*_max_, and its developmental drivers. Test statistics for SNP associations (EMMAX) with the abaxial (**A**) maximum theoretical anatomical stomatal conductance, *g*_max_, (**B**) stomatal fraction, *f*, (**C**) stomatal density, *d*, (**D**) epidermal pavement cell size, *e*, (**E**) stomatal index, *i*, and (**F**) stomatal size, *s*. Dots represent the top 1% associated SNPs along the five chromosomes (alternate gray and black dots represent chromosomes). Red lines represent the genome wide significance threshold with Bonferroni correction.

**Figure S3.**

Genome wide association mapping of variation in adaxial anatomical maximum stomatal conductance, *g*_max_ and its developmental drivers. Test statistics for SNP associations (EMMAX) with the adaxial (**A**) maximum theoretical anatomical stomatal conductance, *g*_max_, (**B**) stomatal fraction, *f*, (**C**) stomatal density, *d*, (**D**) epidermal pavement cell size, *e*, (**E**) stomatal index, *i*, and (**F**) stomatal size, *s*. Dots represent the top 1% associated SNPs along the five chromosomes (alternate gray and black dots represent chromosomes). Red lines represent the genome wide significance threshold with Bonferroni correction. The only significant SNP (*i*_ada_, chromosome 3), is located in a non-coding region of the genome (NIH Genome Data Viewer).

Figure S4.

(A) Relationship of the mean annual temperature, MAT, and (B) the precipitation of the growing season, P_GS_, with an important life-history trait in Arabidopsis, the flowering time at 16ºC, FT_16_. (C-E) Relationships of the abaxial, adaxial and total maximum theoretical anatomical stomatal conductance, *g*_max_, with FT_16_ (Table S6). Solid lines represent the standardized major axis fit. **p* < 0.05; ***p* < 0.01; ****p* < 0.001.

**References**

Alonso-Blanco, C., Andrade, J., Becker, C., Bemm, F., Bergelson, J., Borgwardt, K.M., *et al.* (2016). 1,135 Genomes reveal the global pattern of polymorphism in *Arabidopsis thaliana*. *Cell*, 166, 481–491.

Buckley, T.N. & Diaz-Espejo, A. (2015). Partitioning changes in photosynthetic rate into contributions from different variables: Partitioning changes in photosynthesis. *Plant Cell Environ*, 38, 1200–1211.

Camargo, M.A.B. & Marenco, R.A. (2011). Density, size and distribution of stomata in 35 rainforest tree species in Central Amazonia. *Acta Amaz.*, 41, 205–212.

Carignato, A., Vázquez-Piqué, J., Tapias, R., Ruiz, F. & Fernández, M. (2019). Variability and Plasticity in Cuticular Transpiration and Leaf Permeability Allow Differentiation of Eucalyptus Clones at an Early Age. *Forests*, 11, 9.

Dittberner, H., Korte, A., Mettler-Altmann, T., Weber, A.P.M., Monroe, G. & de Meaux, J. (2018). Natural variation in stomata size contributes to the local adaptation of water-use efficiency in *Arabidopsis thaliana*. *Mol Ecol*, 27, 4052–4065.

Dow, G.J., Berry, J.A. & Bergmann, D.C. (2014). The physiological importance of developmental mechanisms that enforce proper stomatal spacing in *Arabidopsis thaliana*. *New Phytol*, 201, 1205–1217.

Dunbar‐Co, S., Sporck, M.J. & Sack, L. (2009). Leaf trait diversification and design in seven rare taxa of the Hawaiian *Plantago* radiation. *Int J Plant Sci*, 170, 61–75.

Eden, E., Lipson, D., Yogev, S. & Yakhini, Z. (2007). Discovering Motifs in Ranked Lists of DNA Sequences. *PLoS Comput Biol*, 3, e39.

Eden, E., Navon, R., Steinfeld, I., Lipson, D. & Yakhini, Z. (2009). GOrilla: a tool for discovery and visualization of enriched GO terms in ranked gene lists. *BMC Bioinformatics*, 10, 48.

Feltz, C.J. & Miller, G.E. (1996). An asymptotic test for the equality of coefficients of variation from k populations. *Statist Med*, 15, 647–658.

Fletcher, L.R., Scoffoni, C., Farrell, C., Buckley, T.N., Pellegrini, M. & Sack, L. (2022). Testing the association of relative growth rate and adaptation to climate across natural ecotypes of *Arabidopsis*. *New Phytologist*, 236, 413–432.

Franks, P.J. & Beerling, D.J. (2009). Maximum leaf conductance driven by CO _2_ effects on stomatal size and density over geologic time. *Proc. Natl. Acad. Sci. U.S.A.*, 106, 10343–10347.

Franks, P.J., Drake, P.L. & Beerling, D.J. (2009a). Plasticity in maximum stomatal conductance constrained by negative correlation between stomatal size and density: an analysis using *Eucalyptus globulus*. *Plant Cell Environ*.

Franks, P.J., Drake, P.L. & Beerling, D.J. (2009b). Plasticity in maximum stomatal conductance constrained by negative correlation between stomatal size and density: an analysis using *Eucalyptus globulus*. *Plant Cell Environ*.

Franks, P.J. & Farquhar, G.D. (2007). The mechanical diversity of stomata and its significance in gas-exchange control. *Plant Physiol*, 143, 78–87.

Gatto, L. & Lilley, K.S. (2012). MSnbase-an R/Bioconductor package for isobaric tagged mass spectrometry data visualization, processing and quantitation. *Bioinformatics*, 28, 288–289.

Grubb, P.J., Grubb, E.A.A. & Miyata, I. (1975). Leaf structure and function in evergreen trees and shrubs of Japanese warm temperate rain forest I. The structure of the lamina. *Bot Mag Tokyo*, 88, 197–211.

Henry, C., John, G.P., Pan, R., Bartlett, M.K., Fletcher, L.R., Scoffoni, C., *et al.* (2019). A stomatal safety-efficiency trade-off constrains responses to leaf dehydration. *Nat Commun*, 10, 3398.

Hetherington, A.M. & Woodward, F.I. (2003). The role of stomata in sensing and driving environmental change. *Nature*.

Hijmans, R.J., Cameron, S.E., Parra, J.L., Jones, P.G. & Jarvis, A. (2005). Very high resolution interpolated climate surfaces for global land areas. *Int J Climatol*, 25, 1965–1978.

Hijmans, R.J. & van Etten, J. (2012). raster: Geographic analysis and modeling with raster data.

Horton, M.W., Hancock, A.M., Huang, Y.S., Toomajian, C., Atwell, S., Auton, A., *et al.* (2012). Genome-wide patterns of genetic variation in worldwide Arabidopsis thaliana accessions from the RegMap panel. *Nat Genet*, 44, 212–216.

Hothorn, T., Bretz, F. & Westfall, P. (2008). Simultaneous inference in general parametric models. *Biom J*, 50, 346–363.

Kardiman, R. & Ræbild, A. (2018). Relationship between stomatal density, size and speed of opening in Sumatran rainforest species. *Tree Physiol*, 38, 696–705.

Krishnamoorthy, K. & Lee, M. (2014). Improved tests for the equality of normal coefficients of variation. *Comput Stat*, 29, 215–232.

Lasky, J.R., Des Marais, D.L., McKAY, J.K., Richards, J.H., Juenger, T.E. & Keitt, T.H. (2012). Characterizing genomic variation of *Arabidopsis thaliana*: the roles of geography and climate. *Mol Ecol*, 21, 5512–5529.

Liu, C., He, N., Zhang, J., Li, Y., Wang, Q., Sack, L., *et al.* (2018). Variation of stomatal traits from cold temperate to tropical forests and association with water use efficiency. *Funct Ecol*, 32, 20–28.

Machado, R., Loram‐Lourenço, L., Farnese, F.S., Alves, R.D.F.B., De Sousa, L.F., Silva, F.G., *et al.* (2021). Where do leaf water leaks come from? Trade‐offs underlying the variability in minimum conductance across tropical savanna species with contrasting growth strategies. *New Phytologist*, 229, 1415–1430.

Marwick, B. & Krishnamoorthy, K. (2019). cvequality: tests for the equality of coefficients of variation from multiple groups.

McElwain, J.C., Yiotis, C. & Lawson, T. (2016). Using modern plant trait relationships between observed and theoretical maximum stomatal conductance and vein density to examine patterns of plant macroevolution. *New Phytol*.

McKown, A.D., Akamine, M.E. & Sack, L. (2016). Trait convergence and diversification arising from a complex evolutionary history in Hawaiian species of Scaevola. *Oecologia*, 181, 1083–1100.

Medeiros, C.D., Scoffoni, C., John, G.P., Bartlett, M.K., Inman‐Narahari, F., Ostertag, R., *et al.* (2019). An extensive suite of functional traits distinguishes Hawaiian wet and dry forests and enables prediction of species vital rates. *Funct Ecol*, 33, 712–734.

Muchow, R.C. & Sinclair, T.R. (1989). Epidermal conductance, stomatal density and stomatal size among genotypes of  *Sorghum bicolor*  (L.) Moench. *Plant Cell Environ*, 12, 425–431.

Murray, M., Soh, W.K., Yiotis, C., Spicer, R.A., Lawson, T. & McElwain, J.C. (2020). Consistent Relationship between Field-Measured Stomatal Conductance and Theoretical Maximum Stomatal Conductance in C _3_ Woody Angiosperms in Four Major Biomes. *International Journal of Plant Sciences*, 181, 142–154.

Ochoa, M.E., Henry, C., John, G., Medeiros, C., Pan, R., Scoffoni, C., *et al.* (2024). Pinpointing the causal influences of stomatal anatomy and behavior on minimum, operational, and maximum leaf surface conductance. *Plant Physiol*.

Ohsumi, A., Kanemura, T., Homma, K., Horie, T. & Shiraiwa, T. (2007). Genotypic variation of stomatal conductance in relation to stomatal density and length in rice (*Oryza sativa* L.). *Plant Prod Sci*, 10, 322–328.

Pillitteri, L.J. & Dong, J. (2013). Stomatal development in Arabidopsis. *The Arabidopsis Book*, 26.

Purcell, S., Neale, B., Todd-Brown, K., Thomas, L., Ferreira, M.A.R., Bender, D., *et al.* (2007). PLINK: A Tool Set for Whole-Genome Association and Population-Based Linkage Analyses. *The American Journal of Human Genetics*, 81, 559–575.

R Core Team. (2020). R: a language and environment for statistical computing.

Sack, L. & Buckley, T.N. (2016). The developmental basis of stomatal density and flux. *Plant Physiol*.

Sack, L., Cowan, P.D., Jaikumar, N. & Holbrook, N.M. (2003). The ‘hydrology’ of leaves: co-ordination of structure and function in temperate woody species. *Plant Cell Environ*.

Sartori, K., Vasseur, F., Violle, C., Baron, E., Gerard, M., Rowe, N., *et al.* (2019). Leaf economics and slow-fast adaptation across the geographic range of Arabidopsis thaliana. *Sci Rep*, 9, 10758.

Sokal, R.R. & Rohlf, F.J. (2012). Biometry: the principles and practice of statistics in biological research.

Taylor, S.H., Franks, P.J., Hulme, S.P., Spriggs, E., Christin, P.A., Edwards, E.J., *et al.* (2012). Photosynthetic pathway and ecological adaptation explain stomatal trait diversity amongst grasses. *New Phytologist*, 193, 387–396.

Trabucco, A. & Zomer, R.J. (2018). Global Aridity Index and Potential Evapo-Transpiration (ET0) Climate Database v2. CGIAR Consortium for Spatial Information (CGIAR-CSI).

Turner. (2018). qqman: an R package for visualizing GWAS results using Q-Q and manhattan plots. *JOSS*, 3, 731.

Wang, R., Yu, G., He, N., Wang, Q., Zhao, N., Xu, Z., *et al.* (2015). Latitudinal variation of leaf stomatal traits from species to community level in forests: linkage with ecosystem productivity. *Sci Rep*.

Yin, Q., Tian, T., Kou, M., Liu, P., Wang, L., Hao, Z., *et al.* (2020). The relationships between photosynthesis and stomatal traits on the Loess Plateau. *Glob Ecol Conserv*, 23, e01146.

Yu, J., Pressoir, G., Briggs, W.H., Vroh Bi, I., Yamasaki, M., Doebley, J.F., *et al.* (2006). A unified mixed-model method for association mapping that accounts for multiple levels of relatedness. *Nat Genet*, 38, 203–208.

Zhou, X., Carbonetto, P. & Stephens, M. (2013). Polygenic Modeling with Bayesian Sparse Linear Mixed Models. *PLoS Genet*, 9, e1003264.

Zhou, X. & Stephens, M. (2012). Genome-wide efficient mixed-model analysis for association studies. *Nat Genet*, 44, 821–824.
