# Supplementary material for: Dovetailing the Genetic, Developmental and Functional Bases of the Adaptation of Stomatal Traits to Climate
Source: Ecol Lett. 2026 Sep 16;29(9):e70453. doi: 10.1111/ele.70453 (PMC13579612; doi:10.1111/ele.70453)
Supplement: Supplementary file 2 [file ELE-29-0-s003.docx]

***Ecology Letters***

**Appendix S3**

**Supplementary Information for:**

**Dovetailing the genetic, developmental and functional bases**

**of the adaptation of stomatal traits to climate**

**Authors:** Camila D. Medeiros^1,2*^, Thomas N. Buckley^3^, Kevin Sartori^4^, Cyrille Violle^4^, Denis Vile^5^, François Vasseur^4,5^, Leila R. Fletcher^6^, Matteo Pellegrini^7^, Etienne Baron^4^, Elena Kazakou^4^, Lawren Sack^1*^

**Assumptions and limitations of the single common garden design for assessing adaptation to climate including Tables S8-S10**

**Assumptions and limitations of the single common garden design for assessing adaptation to climate**

Our study focuses on a single common garden with benign conditions, which is one of several major approaches currently used for assessing the adaptive basis of trait-climate associations (Tables S8 and S9; (Aitken *et al.* 2024; De Villemereuil *et al.* 2016; Schwinning *et al.* 2022). Notably, there is strong variation across fields of science, and even within evolution and ecology in the definition of biological adaptation, not least because the word can refer to both patterns observed, and underlying processes (Gardner 2009; Kawecki & Ebert 2004; Leimu & Fischer 2008; Simonet 2010). Further, there is strong variability in the specific definition of adaptation that can be tested and inferred from the range of common experimental designs for assessing trait-climate adaptation across and within species (Tables S8 and S9 and refences therein). For example, considering a universal definition of adaptation as “if a being lives and reproduces in its environment, it is because it has adjusted its biological functions to its external conditions” (Simonet 2010), one might support this by examining plants measured in the field (Carlquist 2001; Edwards 2006; Skelton *et al.* 2021; Tudor *et al.* 2024), whereas to prove a definition of adaptation that involves genetic differences, one requires a common garden (see example studies surveyed in Table S8). Yet another, far narrower definition of adaptation, with potentially deeper insights into mechanisms of evolution and ecology, is that local adaptation requires a phenotype or genotype to provide a home-site advantage in fitness over other plants in its environment, and a disadvantage outside their home site (Kawecki & Ebert 2004; Leimu & Fischer 2008). Assessing this definition of adaptation requires multiple common gardens or reciprocal transplants, and, for an even narrower definition, a demonstration that fitness measures are associated with the studied phenotype (Anderson *et al.* 2015; Cui *et al.* 2018; Ostrowsky *et al.* 2026) (Table S9). Notably, generally, logistics has prevented any study to our knowledge from using the complete set of approaches (Table S8). It is thus important to recognize and acknowledge the limits of the meaning of adaptation that can be inferred from any study design, its benefits, and its assumptions if it is to be extended to more specific understanding of adaptation.

A rich and ongoing literature attests to the power of the single common garden approach in demonstrating relationships between traits with potential fitness effects and source environment across species and ecotypes (Table S9). By controlling for environmental variation, one measures phenotypic variation with a genetic basis, that is not influenced by plasticity with respect to the environment, or by genotype × environment interactions that would result in variation in plasticity across genotypes from different environments (Aitken *et al.* 2024; Baird *et al.* 2021; Blanco-Sánchez *et al.* 2023; Britton *et al.* 2026; Cavender‐Bares *et al.* 2005; Clausen *et al.* 1940; Cordell *et al.* 1998; Dittberner *et al.* 2018; Fasanella *et al.* 2024; Fisher *et al.* 2007; Guo *et al.* 2022; Huxman *et al.* 2022; Jacob *et al.* 2025; Kitayama *et al.* 1997; Martin *et al.* 2007; Meng *et al.* 2025; Pan *et al.* 2025; Ramirez-Valiente *et al.* 2010; Ramírez‐Valiente *et al.* 2022; Ramírez-Valiente & Cavender-Bares 2017; Schwinning *et al.* 2022; Scoffoni *et al.* 2016; Tsvuura & Ward 2022). This approach enables tests of adaptation defined as “a species or ecotype in its native climate being adjusted in its biological functions to its external conditions, with heritable phenotypic variation (genetic determination)”. Narrower and possibly deeper definitions of adaptation, such as that the phenotypic variation is linked with a home site advantage in fitness would require additional studies (Table S9). We discuss here the assumptions involved for the latter definition to apply based on our study system.

A first assumption, typical in a large literature on adaptation using single common gardens (Baird *et al.* 2021; Britton *et al.* 2026; Cavender‐Bares *et al.* 2005; Cordell *et al.* 1998; Dittberner *et al.* 2018; Fasanella *et al.* 2024; Fisher *et al.* 2007; Guo *et al.* 2022; Jacob *et al.* 2025; Kitayama *et al.* 1997; Martin *et al.* 2007; Meng *et al.* 2025; Pan *et al.* 2025; Ramirez-Valiente *et al.* 2010; Ramírez-Valiente & Cavender-Bares 2017; Scoffoni *et al.* 2016; Tsvuura & Ward 2022) (Table S8), is that if an ecotype or population was sampled in an environment in which it is common, then it had high fitness there, and that the trait studied contributed to that fitness (Lankau & Keymer 2018; Leimu & Fischer 2008). However, multiple factors in principle may result in species and ecotypes occurring in a site being to a greater or lesser degree maladapted to their climate (Table S10); including population structure effects and disequilibria (Aitken *et al.* 2024; Sork *et al.* 2013), mismatch between time of sampling and time since occupation of a given habitat (Anderson *et al.* 2025; Browne *et al.* 2019; Matyas 1994), disturbances (Malíková *et al.* 2016; Schierenbeck 2017), competition and other biotic effects (Hayashi & Kraft 2025), gene flow (Legrand *et al.* 2017), and adaptation lag (Aguirre-Gutiérrez *et al.* 2025; Matyas 1994; Wilczek *et al.* 2014a). Further, in principle the ecotype may be adapted not to climate per se across sampled environments, but instead to other variables covarying with climate, or to unmeasured biotic or abiotic factors. Additionally, the relationship assessed may be affected by uncertainty in the measured climate variables, and, indeed, the degree that the climate variables correspond to the microclimate experienced by the plant in the field. While population structure effects can be accounted for using kinship analyses, as implemented in our study (Yu *et al.* 2006), the other factors are not easily addressed. Definitive proof of adaptation in the sense of a home site fitness advantage (and even a *non-home site* *disadvantage*) requires reciprocal transplant experiments or multiple common gardens, and tests for genetic adaptation using evolutionary models that can account for neutrality (De Villemereuil *et al.* 2018, 2022; Johnson *et al.* 2022). Importantly, for *Arabidopsis thaliana*, numerous studies using multiple common gardens have repeatedly found ecotypes native to different climates had home-site advantage in their native climates with fitness assessed using fecundity (Ågren & Schemske 2012; Hamilton *et al.* 2015; Rutter & Fenster 2007; Wilczek *et al.* 2014a), or mapping of fitness-associated loci with climate (Fournier-Level *et al.* 2011). Similar studies have found similarly for other plant species (Lortie & Hierro 2022; Ramirez-Valiente *et al.* 2010; Ramírez‐Valiente *et al.* 2022).

A second assumption, on testing adaptation using single common gardens, is that genetic differences are a major source of beneficial variation in the trait, because plasticity and gene-environment interactions, which could contribute to performance advantages, would not be discerned in the common garden. Thus, in the common garden, in benign conditions, a plant native to a more stressful environment would not manifest, via trait plasticity, certain differences that may contribute to its advantageous phenotype in the stressful environment (Legrand *et al.* 2017). Or, alternatively, in the common garden, differences may manifest that are stronger than would occur in the field, if plasticity would result in environmental canalization (i.e., tendency to similar phenotypes across environments (Wang & Zhou 2021). To quantify such plasticity and its possible advantage for performance or fitness across climates, multiple common gardens or reciprocal transplants are necessary (Table S9). However, we note that in general, the finding of a significant trait-climate association in a common garden would result in a *conservative* test of a tendency for adaptation using a definition that includes the beneficial effects of plasticity, as the garden excludes plasticity that might increase fitness in stressful environments. Here, to test the overall validity of the common garden approach to indicate trends that would be found if also including plasticity and gene-environment interactions in the field, we surveyed 13 studies in the literature that enable comparison of trait-climate associations determined in a benign common garden with those found in across environments in other common gardens or in the field, based on searches using online databases (Table S8, column (5)). We found that 12/13 (92%) of studies of phenotypic traits supported congruent trait-climate associations in a single benign common garden and in other common gardens or field transplant or field populations under extreme conditions; the one exception was for populations of non-native invasive species which showed no trait-climate association in the common garden like that observed in the field plants (Ebeling *et al.* 2011) (Table S8). This validation, for the cases of species or ecotypes native to their home sites, attests to the robustness of the approach of comparing plant in a single benign common garden to resolve trait relationships with native climate observed in field grown plants across a climatic range of habitats or common gardens.

A third assumption, typical in the literature on adaptation as assessed by trait-climate correlations observed in a common garden is that the phenotypic trait under study is “adaptive”, i.e., contributes to the fitness of the species (Caruso *et al.* 2020). In principle, a trait could be studied to ensure its correlation or direct mechanistic contribution to measures of fitness such as survival, fecundity, seed viability, etc. However, a large literature of common garden studies do not implement these tests, and assume that the advantage provided by the trait to the plant, as assessed in other studies, would contribute to fitness and thus to adaptation in the home site (Table S8, column (6)), and, indeed, several studies have shown that simple phenotypic traits are associated with fitness advantages under different climates as expected (Table S8, columns (6) and (7); Table 9). In our study, we focused on stomatal traits, and especially the maximum anatomical stomatal conductance, or *g*_max_. Directly testing the contribution of *g*_max_ differences to fitness, i.e., its linkage with components of fitness such as fecundity, seed viability, etc., or with variation in fitness across climates, was outside the scope of our paper. Here we draw on rich literature that established linkages of stomatal traits with adaptation across species and ecotypes, by considering *g*_max_ as a proxy for gas exchange potential. The strongest evidence supporting this assumption of our study is that *g*_max_ strongly predicted the operating stomatal conductance measured with leaf gas exchange systems (*g*_op_) across *Arabidopsis thaliana* genotypes under low CO_2_, high humidity, and high red and blue light (Dow *et al.* 2014). Notably, under typical conditions, *g*_max_ values tend to be much higher than *g*_op_ (Feild *et al.* 2011; McElwain *et al.* 2016; Ochoa *et al.* 2024; Sack & Buckley 2016) for several reasons. First, the effective area of the stomatal pore (*a*’) is typically smaller than the anatomical maximum *a*_max_, depending on external and internal conditions, e.g., irradiance and leaf hydration state (Crawford *et al.* 2025; Ochoa *et al.* 2024). Second, features other than stomatal pore area, such as the anatomy of the intercellular airspaces and leaf surface features such as hairs or papillae surrounding the stomata, may also influence the overall diffusive conductance and therefore gas exchange (Feild *et al.* 2011; Hassiotou *et al.* 2009; Kenzo *et al.* 2008; Maricle *et al.* 2009; Roth-Nebelsick 2007). The anatomical *g*_max_ is therefore a theoretical value estimating the maximum stomatal diffusion capacity, and like other theoretical physiological variables, such as photosynthetic parameters including the maximum carboxylation rate (*V*_cmax_), is not generally reached in practice, but is useful for generating hypotheses regarding the capacity for stomatal diffusion in various domains, and as a robust parameter to assess photosynthetic potential such as comparisons of genotypes or species, functional types, or trends in evolutionary time (de Boer *et al.* 2016; Doheny-Adams *et al.* 2012; Franks *et al.* 2009; McElwain *et al.* 2016; Taylor *et al.* 2012), especially because it relies on structural characteristics and is thus less dependent on differences in irradiance and time of measurement (Lawson & Blatt 2014). Yet, as mentioned above, *g*_max_ correlates across diverse species and Arabidopsis genotypes with *g*_op_ and light-saturated photosynthetic rate (Dow *et al.* 2014; McElwain *et al.* 2016; Ochoa *et al.* 2024). Given that stomatal conductance is a primary limitation on photosynthesis, all else being equal, it would scale up to the determination of plant relative growth rates (RGR) and even ecosystem productivity (Quero *et al.* 2008; Wang *et al.* 2015). Notably, while some studies of mutants and wild plants show that stomatal density and *g*_max_ can predict photosynthetic productivity (e.g., (Caine *et al.* 2019), others do not, because stomatal aperture can compensate for shifts in stomatal density (e.g., (Büssis *et al.* 2006). Further, the scaling up of stomatal traits to leaf photosynthetic capacity, and thereby to plant growth, involves several steps with potential decoupling. Growth, like fitness, is a higher level variable that will depend on multiple lower level variables, a “many-to-one” mapping situation, such that even though the effects of lower-level variables scale up when all else is equal, individual ones may not when many traits co-vary, resulting in alternative pathways to high fitness (Marks & Lechowicz 2006; Yang *et al.* 2018). In particular, theory and experiments show that the translation of photosynthetic rate into relative growth rate (RGR) depends also on biomass allocation and leaf morphology (Fletcher *et al.* 2022; Liu *et al.* 2020; Quero *et al.* 2008; Weraduwage *et al.* 2015; Yavari *et al.* 2021). Importantly, even though RGR can be decoupled from leaf-scale photosynthesis, in comparisons across species or ecotypes, photosynthetic rate is still the mechanistic driver of RGR. Thus, for example, while a stress-resistant ecotype with a high photosynthetic rate but low leaf allocation (and, say, high root allocation), may thus have a low RGR, its RGR is not as low as it would be if it did not have a high photosynthetic rate. Thus, overall, we consider *g*_max_ to be a potentially adaptive trait, such that when high, it enables more rapid photosynthesis and therefore growth, and when low, it potentially reduces water requirements and increases intrinsic water use efficiency (Gray & Dunn 2024). Further, stomatal traits including *g*_max_ may have additional adaptive functions, such as temperature regulation or pathogen resistance (Busby *et al.* 2026). Supporting our assumption, stomatal density and *g*_max_ are widely-recognized targets for crop improvement (Ferguson *et al.* 2024; Gray & Dunn 2024).

While our experimental design, given these assumptions, enables us to test the adaptive nature of the associations of stomatal traits with native climate, given our definition of adaptation, we recognize that additional experiments would be needed to support adaptation in a narrower sense, and to test assumptions of the contributions of *g*_max_ to fitness that are widespread in the literature. Our approach, testing dovetailing frameworks of development, function and climate adaptation would be applicable in such future studies, and thus can help guide that research and open up additional avenues for expanded understanding of trait adaptation to climate.

**Table S8.** Survey of studies of trait-climate adaptation using different common approaches (column 2); no attempt was made to collect all studies using single or multiple common gardens and/or reciprocal transplant study designs. We emphasize in column (3) the number and range of studies that inferred “adaptation” from a single common garden experiment and other designs (see Table S9 for inference enabled by study designs), in column (5) multiple studies enabling comparison of trait-climate associations determined across multiple common gardens, or in the field across environments, and in columns (6) and (7) studies that further validated the the phenotype of interest was linked to measures of fitness across environments.

| **(1) Study** | **(2) Field plants, single common garden, multiple common gardens or reciprocal transplant experiments** | **(3) Does the study infer “adaptation” from trait-environment associations** | **(4) Comparative context** | **(5) Trait-environment correlation in common garden matches in direction that in field-grown or reciprocal transplant or multiple common gardens in different environments** | **(6) Does the study include a measure of fitness, potentially validating the assumption of home-site advantage?** | **(7) Trait-environment correlation in common garden matched with fitness advantage in home environment, supporting phenotype-fitness association?** |
| --- | --- | --- | --- | --- | --- | --- |
| (Edwards 2006) | Field plants | Yes | Seven species of *Pereskia* (Cactaceae) | NA | No | NA |
| (Skelton *et al.* 2021) | Field plants | Yes | 19 *Quercus* species sampled within their native range of distribution | NA | No | NA |
| (Carlquist 1977) | Field plants | Yes | Species of multiple habitats across SW Australia | NA | No | NA |
| (Tudor *et al.* 2024) | Field plants | Yes | Five populations of *Stylidium* *hispidum* sampled along a latitudinal and climatic gradient in Australia | NA | No | NA |
| (Cavender‐Bares *et al.* 2005) | Single common garden | Yes | Four *Quercus* species sourced from different climatic origins and grown in a common garden | NA | No | NA |
| (Ramirez-Valiente *et al.* 2010) | Single common garden | Yes | 13 populations of *Quercus suber* sourced from diverse climates and grown in a common garden | NA | Yes, growth | NA |
| (Scoffoni *et al.* 2016) | Single common garden | Yes | 30 *Viburnum* species from different climatic origins grown in a common garden | NA | No | NA |
| (Ramírez-Valiente & Cavender-Bares 2017) | Single common garden | Yes | Five populations of *Quercus oleoides* across an aridity gradient | NA | Yes, absolute and relative growth rates | NA |
| (Dittberner *et al.* 2018) | Single common garden | Yes | 330 genotypes of Arabidopsis grown in a growth chamber | NA | No | NA |
| (Guo *et al.* 2022) | Single common garden | Yes | Phenology of *Picea mariana* saplings originating from five provenances growing in a common garden | NA | No | NA |
| (Fasanella *et al.* 2024) | Single common garden | Yes | *Austrocedrus chilensis* sourced from the extreme ends of a precipitation gradient (dry vs. humid populations) and grown in a common garden | NA | No | NA |
| (Pan *et al.* 2025) | Single common garden | Yes | 58 *Eucalyptus* species sampled from different climatic origins grown in a greenhouse | NA | No | NA |
| (Jacob *et al.* 2025) | Single common garden | Yes | Eight populations of *Themeda triandra* sourced from different climatic origins grown in a greenhouse | NA | No | NA |
| (Meng *et al.* 2025) | Single common garden | Yes | 1300 genotypes of *Populus trichocarpa* sourced from diverse climates and grown in a common garden | NA | Yes; relative growth rate, height | NA |
| (Kitayama *et al.* 1997) | Field plants and single common garden | Yes | Populations of *Metrosideros polymorpha* trees, with glabrous and pubescent leaf phenotypes native across an altitudinal gradient in Hawaii | Yes, for leaf pubescence | No | NA |
| (Cordell *et al.* 1998) | Field plants and single common garden | Yes | *Metrosideros polymorpha* trees along an altitudinal gradient, and different soil ages | Yes, for carbon isotope ratio, leaf N concentration, leaf mass per area and leaf and tissue thicknesses vs elevation* | No | NA |
| (Fisher *et al.* 2007) | Field plants and single common garden | Yes | *Metrosideros polymorpha* genotypes across an elevation gradient | Yes, for mean vessel diameter | No | NA |
| (Martin *et al.* 2007; Martin & Asner 2009) | Field plants and single common garden | Yes in both studies | *Metrosideros polymorpha* trees sampled from 14 different populations across an elevation gradient grown in a common garden, and from 13 different populations across an elevation gradient in Hawaii | Yes, for 8/8 measured leaf chemical and optical properties | No | NA |
| (Baird *et al.* 2021) | Field plants and single common garden | Yes | 27 diverse C_3_ and C_4_ grass species grown in a greenhouse and 912 C_3_ and 840 C_4_ species from databases | Yes, for leaf area | No | NA |
| (Britton *et al.* 2026) | Field plants and single common garden | Yes | 454 eucalypt taxa from diverse climate origins grown in a common garden and measured across their native range of distribution (data extracted from a database) | Yes, for 7/8 traits with MAP | No | NA |
| (Givnish *et al.* 2004; Givnish & Montgomery 2014) | Field plants and four common gardens | Yes in both studies | 11 species of Hawaiian lobeliads growing in their native habitat, and 6 species grown in a common garden | Yes, for photosynthetic rate vs irradiance (similar trends for plants in the field and in each common garden) | No | NA |
| (Cavender-Bares & Ramírez-Valiente 2017) | Multiple common gardens | Yes | *Quercus oleioides* populations across an aridity gradient grown as seedlings in a greenhouse common garden, and two field common gardens | Yes, for specific leaf area | No | NA |
| (De Villemereuil *et al.* 2018) | Multiple common gardens | Yes | *Arabis alpina* populations across elevations | NA | Yes; growth, survival, fruit length | Yes |
| (Fontes *et al.* 2022) | Multiple common gardens | Yes | 18 oak species grown in three common gardens spanning contrasting climates | NA | No | NA |
| (Smith *et al.* 2023) | Multiple common gardens | Yes | 10 species of *Eucalyptus* native across an aridity gradient in Australia grown in four common gardens across the aridity gradient | Yes, for 47/53 (89%) of ecophysiological variables measured | Yes; survival |  |
| (Martínez-Sancho *et al.* 2025) | Multiple common gardens | Yes | Variability in functional traits of 9 and 11 provenances of *Quercus petraea* and *Fagus sylvatica* grown in four common gardens across their distribution areas | NA | No | NA |
| (Ramírez‐Valiente *et al.* 2022) | Meta-analysis of studies on 21 common gardens of Mediterranean oaks and pines | Yes | Meta-analysis of published studies on functional traits and fitness of *Pinus* sp. and *Quercus* sp. from Mediterranean climates growing in common gardens | Yes, for 21/50 (42%) of the correlations tested among survival and height with climate variables* | Yes; survival rate and height | Yes |
| (Tsvuura & Ward 2022) | Single common garden and reciprocal transplant | Yes | Two neighboring population of tree species *Vachellia karoo* grown under greenhouse conditions and reciprocally transplanted | Yes, similar values for leaf and plant morphology in the common garden and reciprocal transplant sites | No | NA |
| (Anderson *et al.* 2015) | Three common gardens and reciprocal transplant | Yes | Different populations of forb species *Boechera stricta* sampled across its range of distribution in an elevation gradient in the Colorado Rocky Mountains | Yes, herbivory susceptibility was similar across multiple common gardens | Yes; survival, lifetime fitness, germination success | Yes |
| (Ebeling *et al.* 2011) | Three common gardens and reciprocal transplant | No | 20 invasive populations of invasive species *Buddleja davidii* grown in three common gardens with different climates across Europe and reciprocally transplanted | No, no correlation of traits with climate of origin of the population | Yes; number of inflorescences, length of inflorescences | No |
| (Bowman *et al.* 2008) | Reciprocal transplant | Yes | 15 populations of a herb species (*Lychnis flos-cuculi)* replanted/transplanted between 15 grasslands in Switzerland | NA | Yes; number of rosettes per plant, survival and damage by herbivory | Yes |
| (Ostrowsky *et al.* 2026) | Reciprocal transplant | Yes | *Quercus macrocarpa* sampled from across a climatic range | Yes, leaf lobing differences among populations were similar at the transplant sites | Yes; based on survival, seed size and initial height | Yes |
| (Cui *et al.* 2018) | Reciprocal transplant | Yes | Reciprocal transplant of turfs of alpine species *Viola biflora* L. var. *rockiana* between the central parts of its range and the warmer range margin | NA | Yes; population growth rate, growth, fecundity, survival and clonality | Yes |

* Traits in column (5) that were similar across gardens and field sites were those that rely on structural characteristics subject to important internal control in their development in the plant and thus would be expected to reflect genetic or plastic variation (including leaf size, nutrient composition, leaf mass per area, etc.). Measures that did not relate to species' native climate consistently when plants were measured across sites included gas exchange or under ambient conditions, plant height, and plant survival; these variables likely depend strongly on differences in irradiance and time of measurement, or on site-specific soil factors or disturbance or herbivory much more than according to the genetic or plastic variation of the plants.

**Table S9.** Approaches for studying trait-climate adaptation, with example studies (noting that there are a great many that could be reviewed in each category), and the definition of adaptation that is typically tested or inferred [after (Aitken *et al.* 2024; Sork 2017)]. The “adaptation” that can be tested or inferred from these approaches starts very broad, and becomes narrower and possibly provides deeper evolutionary insights, as designs progress from field sampling to simple common garden, to multiple common gardens and reciprocal transplants, and even narrower and mechanistically deeper definitions can be tested if fitness measures are additionally made.

| **Design** | **Factors influencing phenotype-climate association** | **Inference of “adaptation” possible without fitness measures** | **Inference of “adaptation” possible if fitness is measured** |
| --- | --- | --- | --- |
| Plants sampled in the field | Genetic, plasticity to environment, gene-by-environment interactions | Species or ecotype in its native climate has adjusted its biological functions to its external conditions (Edwards 2006; Givnish *et al.* 2004) | NA |
| Single common garden in benign conditions | Genetic differences | Species or ecotype in its native climate has adjusted its biological functions to its external conditions, and the phenotypic variation is  genetically determined (Baird *et al.* 2021; Givnish & Montgomery 2014) | NA |
| Multiple common gardens with different environmental conditions | Genetic differences + plasticity that can be quantified + gene-by-environment interactions that can be quantified | Species or ecotype in its native climate has adjusted its biological functions to its external conditions, and the phenotypic variation is  genetically determined, and  plasticity and gene-environment associations can further contribute to benefit in given climates (Fontes *et al.* 2022; Smith *et al.* 2023) | Species or ecotype in its native climate has adjusted its biological functions to its external conditions, and the phenotypic variation is  genetically determined, and  plasticity and gene-environment associations can further contribute to benefit in given climates; tests of the association of fitness with the trait validate adaptation further, and, additionally one may establish that, the phenotype is associated with a “home site advantage” in fitness, and a disadvantage in their non-home site(s) (Anderson *et al.* 2015) |
| Reciprocal field transplants* | “ | “ (Tsvuura & Ward 2022) | “ (Cui *et al.* 2018; Ostrowsky *et al.* 2026) |

*As for multiple common gardens, but without the control of additional factors that influence field-growth.

**Table S10.** Factors contributing to maladaptation, i.e., fitness and fitness-related phenotypic traits being mismatched from their home site.

| **Sources of maladaptation to climate** | **Rationale** |
| --- | --- |
| Timing mismatch | If a population invaded and persisted at the sampling site for a too-short period to be optimized there, the genes may not show evidence of local adaptation (Anderson *et al.* 2025; Browne *et al.* 2019; Matyas 1994) |
| Disturbance | The genes may reflect adaptation to intense and/or chronic disturbance rather than local climate adaptation (Malíková *et al.* 2016). Human-mediated hybridization can also increase the gene-adaptation mismatch as it may cause homogenization of populations and lead to reduced evolutionary flexibility if locally adapted genes become lost (Schierenbeck 2017) |
| Competition and other biotic effects | The relative fitness of species or populations in given environments may be influenced by their specific competitors or pathogens in the field (Hayashi & Kraft 2025) |
| Barrier to dispersal | The phenotype of a geographically restricted population may be mismatched from its local environment due to genetic drift or not enough time to optimize to the new conditions after the establishment of the barrier (Legrand *et al.* 2017). |
| Gene flow | Gene flow may disrupt the process of optimization if the individuals immigrating into a population have phenotypes that do not match the environmental conditions of the new habitat (Legrand *et al.* 2017) |
| Adaptation lag | The climate optima of local populations may not keep pace with rapid climate change. The population average of a fitness-related trait for a locally adapted population may be lower than the average of other populations introduced from other environments due to a phenomenon termed as adaptation lag. Selection may not track environmental changes fast enough, and traits may be better correlated with historical climate (Aguirre-Gutiérrez *et al.* 2025; Mátyás 1994; Wilczek *et al.* 2014b) |

**References**

Ågren, J. & Schemske, D.W. (2012). Reciprocal transplants demonstrate strong adaptive differentiation of the model organism *Arabidopsis thaliana* in its native range. *New Phytologist*, 194, 1112–1122.

Aguirre-Gutiérrez, J., Díaz, S., Rifai, S.W., Corral-Rivas, J.J., Nava-Miranda, M.G., González-M, R., *et al.* (2025). Tropical forests in the Americas are changing too slowly to track climate change. *Science*, 387, eadl5414.

Aitken, S.N., Jordan, R. & Tumas, H.R. (2024). Conserving Evolutionary Potential: Combining Landscape Genomics with Established Methods to Inform Plant Conservation. *Annual Review of Plant Biology*, 75, 707–736.

Anderson, J.T., DeMarche, M.L., Denney, D.A., Breckheimer, I., Santangelo, J. & Wadgymar, S.M. (2025). Adaptation and gene flow are insufficient to rescue a montane plant under climate change. *Science*, 525–531.

Anderson, J.T., Perera, N., Chowdhury, B. & Mitchell-Olds, T. (2015). Microgeographic Patterns of Genetic Divergence and Adaptation across Environmental Gradients in *Boechera stricta* (Brassicaceae). *Am Nat*, 186, S60–S73.

Baird, A.S., Taylor, S.H., Pasquet-Kok, J., Vuong, C., Zhang, Y., Watcharamongkol, T., *et al.* (2021). Developmental and biophysical determinants of grass leaf size worldwide. *Nature*, 592, 242–247.

Blanco-Sánchez, M., Franks, S.J., Ramos-Muñoz, M., Pías, B., Ramírez-Valiente, J.A., Escudero, A., *et al.* (2023). Contrasting adaptive trait variation in response to drought in two Mediterranean shrubs. *Environmental and Experimental Botany*, 208, 105253.

de Boer, H.J., Price, C.A., Wagner‐Cremer, F., Dekker, S.C., Franks, P.J. & Veneklaas, E.J. (2016). Optimal allocation of leaf epidermal area for gas exchange. *New Phytol*, 210, 1219–1228.

Bowman, G., Perret, C., Hoehn, S., Galeuchet, D.J. & Fischer, M. (2008). Habitat fragmentation and adaptation: a reciprocal replant–transplant experiment among 15 populations of *Lychnis flos‐cuculi*. *Journal of Ecology*, 96, 1056–1064.

Britton, T.G., Halliwell, B., Blackman, C.J., Hartill, G.E., Sumner, E.E., Jacob, V.K., *et al.* (2026). Trait–climate relationships in 454 eucalypt taxa: common garden vs field-derived trends. *New Phytol*, nph.70930.

Browne, L., Wright, J.W., Fitz-Gibbon, S., Gugger, P.F. & Sork, V.L. (2019). Adaptational lag to temperature in valley oak (*Quercus lobata*) can be mitigated by genome-informed assisted gene flow. *Proc Natl Acad Sci USA*, 116, 25179–25185.

Busby, P.E., Apigo, A., Sirova, D., Pérez‐Pazos, E., Gervers, K.A., Neat, A., *et al.* (2026). Do stomatal traits modulate leaf microbiome assembly? *New Phytologist*, nph.70914.

Büssis, D., von Groll, U., Fisahn, J. & Altmann, T. (2006). Stomatal aperture can compensate altered stomatal density in Arabidopsis thaliana at growth light conditions. *Functional Plant Biology*, 33, 1037–1043.

Caine, R.S., Yin, X., Sloan, J., Harrison, E.L., Mohammed, U., Fulton, T., *et al.* (2019). Rice with reduced stomatal density conserves water and has improved drought tolerance under future climate conditions. *New Phytologist*, 221, 371–384.

Carlquist, S. (1977). Ecological factors in wood evolution: a floristic approach. *Am J Bot*, 64, 887–896.

Carlquist, S. (2001). *Comparative Wood Anatomy*. Springer Series in Wood Science. 2nd edn. Springer-­Verlag, Berlin.

Caruso, C.M., Mason, C.M. & Medeiros, J.S. (2020). The Evolution of Functional Traits in Plants: Is the Giant Still Sleeping? *International Journal of Plant Sciences*, 181, 1–8.

Cavender‐Bares, J., Cortes, P., Rambal, S., Joffre, R., Miles, B. & Rocheteau, A. (2005). Summer and winter sensitivity of leaves and xylem to minimum freezing temperatures: a comparison of co‐occurring Mediterranean oaks that differ in leaf lifespan. *New Phytol*, 168, 597–612.

Cavender-Bares, J. & Ramírez-Valiente, J.A. (2017). Physiological Evidence from Common Garden Experiments for Local Adaptation and Adaptive Plasticity to Climate in American Live Oaks (Quercus Section Virentes): Implications for Conservation Under Global Change. In: Gil-Pelegrín, E., Peguero-Pina, J., Sancho-Knapik, D. (eds). In: *Oaks Physiological Ecology. Exploring the Functional Diversity of Genus Quercus L.*, Tree Physiology. Springer, Cham, pp. 107–136.

Clausen, J., Keck, D.D. & Hiesey, W.M. (1940). I. Effect of varied environments on western North American plants. In: *Experimental studies on the nature of species*. Washington, D.C.

Cordell, S., Goldstein, G., Mueller-Dombois, D., Webb, D. & Vitousek, P.M. (1998). Physiological and morphological variation in *Metrosideros polymorpha*, a dominant Hawaiian tree species, along an altitudinal gradient: the role of phenotypic plasticity. *Oecologia*, 113, 188–196.

Crawford, J.D., Mayfield-Jones, D., Fried, G.A., Hernandez, N. & Leakey, A.D.B. (2025). Stomata in-sight: Integrating live confocal microscopy with leaf gas exchange and environmental control. *Plant Physiol*, 199, kiaf600.

Cui, H., Töpper, J.P., Yang, Y., Vandvik, V. & Wang, G. (2018). Plastic Population Effects and Conservative Leaf Traits in a Reciprocal Transplant Experiment Simulating Climate Warming in the Himalayas. *Front Plant Sci*, 9, 1069.

De Villemereuil, P., Gaggiotti, O.E. & Goudet, J. (2022). Common garden experiments to study local adaptation need to account for population structure. *Journal of Ecology*, 110, 1005–1009.

De Villemereuil, P., Gaggiotti, O.E., Mouterde, M. & Till-Bottraud, I. (2016). Common garden experiments in the genomic era: new perspectives and opportunities. *Heredity*, 116, 249–254.

De Villemereuil, P., Mouterde, M., Gaggiotti, O.E. & Till‐Bottraud, I. (2018). Patterns of phenotypic plasticity and local adaptation in the wide elevation range of the alpine plant *Arabis alpina*. *Journal of Ecology*, 106, 1952–1971.

Dittberner, H., Korte, A., Mettler-Altmann, T., Weber, A.P.M., Monroe, G. & de Meaux, J. (2018). Natural variation in stomata size contributes to the local adaptation of water-use efficiency in *Arabidopsis thaliana*. *Mol Ecol*, 27, 4052–4065.

Doheny-Adams, T., Hunt, L., Franks, P.J., Beerling, D.J. & Gray, J.E. (2012). Genetic manipulation of stomatal density influences stomatal size, plant growth and tolerance to restricted water supply across a growth carbon dioxide gradient. *Phil Trans R Soc B*, 367, 547–555.

Dow, G.J., Bergmann, D.C. & Berry, J.A. (2014). An integrated model of stomatal development and leaf physiology. *New Phytol*, 201, 1218–1226.

Ebeling, S.K., Stocklin, J., Hensen, I. & Auge, H. (2011). Multiple common garden experiments suggest lack of local adaptation in an invasive ornamental plant. *J Plant Ecol*, 4, 209–220.

Edwards, E.J. (2006). Correlated evolution of stem and leaf hydraulic traits in *Pereskia* (Cactaceae). *New Phytologist*, 172, 479–789.

Fasanella, M., Souto, C.P., Kitzberger, T. & Premoli, A.C. (2024). Common garden experiments and SNP genotyping at the extremes of a steep precipitation gradient suggest local adaptation in a Patagonian conifer. *Botanical Journal of the Linnean Society*, 206, 14–28.

Feild, T.S., Upchurch, G.R., Chatelet, D.S., Brodribb, T.J., Grubbs, K.C., Samain, M.-S., *et al.* (2011). Fossil evidence for low gas exchange capacities for Early Cretaceous angiosperm leaves. *Paleobiology*, 37, 195–213.

Ferguson, J.N., Schmuker, P., Dmitrieva, A., Quach, T., Zhang, T., Ge, Z., *et al.* (2024). Reducing stomatal density by expression of a synthetic epidermal patterning factor increases leaf intrinsic water use efficiency and reduces plant water use in a C4 crop. *Journal of Experimental Botany*, 75, 6823–6836.

Fisher, J.B., Goldstein, G., Jones, T.J. & Cordell, S. (2007). Wood vessel diameter is related to elevation and genotype in the Hawaiian tree *Metrosideros polymorpha* (Myrtaceae). *Am J Bot*, 94, 709–715.

Fletcher, L.R., Scoffoni, C., Farrell, C., Buckley, T.N., Pellegrini, M. & Sack, L. (2022). Testing the association of relative growth rate and adaptation to climate across natural ecotypes of *Arabidopsis*. *New Phytologist*, 236, 413–432.

Fontes, C.G., Pinto‐Ledezma, J., Jacobsen, A.L., Pratt, R.B. & Cavender‐Bares, J. (2022). Adaptive variation among oaks in wood anatomical properties is shaped by climate of origin and shows limited plasticity across environments. *Funct Ecol*, 36, 326–340.

Fournier-Level, A., Korte, A., Cooper, M.D., Nordborg, M., Schmitt, J. & Wilczek, A.M. (2011). A Map of Local Adaptation in *Arabidopsis thaliana*. *Science*, 334, 86–89.

Franks, P.J., Drake, P.L. & Beerling, D.J. (2009). Plasticity in maximum stomatal conductance constrained by negative correlation between stomatal size and density: an analysis using *Eucalyptus globulus*. *Plant Cell Environ*.

Gardner, A. (2009). Adaptation as organism design. *Biol Lett*, 5, 861–864.

Givnish, T.J. & Montgomery, R.A. (2014). Common-garden studies on adaptive radiation of photosynthetic physiology among Hawaiian lobeliads. *Proc R Soc B*, 281, 20132944.

Givnish, T.J., Montgomery, R.A. & Goldstein, G. (2004). Adaptive radiation of photosynthetic physiology in the Hawaiian lobeliads: light regimes, static light responses, and whole‐plant compensation points. *American J of Botany*, 91, 228–246.

Gray, J. & Dunn, J. (2024). Optimizing Crop Plant Stomatal Density to Mitigate and Adapt to Climate Change. *Cold Spring Harb Perspect Biol*, 16, a041672.

Guo, X., Klisz, M., Puchałka, R., Silvestro, R., Faubert, P., Belien, E., *et al.* (2022). Common‐garden experiment reveals clinal trends of bud phenology in black spruce populations from a latitudinal gradient in the boreal forest. *Journal of Ecology*, 110, 1043–1053.

Hamilton, J.A., Okada, M., Korves, T. & Schmitt, J. (2015). The role of climate adaptation in colonization success in *Arabidopsis thaliana*. *Molecular Ecology*, 24, 2253–2263.

Hassiotou, F., Evans, J.R., Ludwig, M. & Veneklaas, E.J. (2009). Stomatal crypts may facilitate diffusion of CO_2_ to adaxial mesophyll cells in thick sclerophylls. *Plant Cell & Environment*, 32, 1596–1611.

Hayashi, K.T. & Kraft, N.J.B. (2025). Competition contributes to quantitative mismatches between plant fitness and occurrence along environmental gradients. *J Ecol*, 113, 2590–2602.

Huxman, T.E., Winkler, D.E. & Mooney, K.A. (2022). A common garden super‐experiment: An impossible dream to inspire possible synthesis. *Journal of Ecology*, 110, 997–1004.

Jacob, V., Blackman, C.J., Choat, B., Atwell, B., Foo, E., Correa-Lozano, A., *et al.* (2025). Linking drought strategy to climate of origin in a widespread C4 grass species: hydraulic traits, xylem anatomy and stomatal behaviour in *Thameda triandra*.

Johnson, L.C., Galliart, M.B., Alsdurf, J.D., Maricle, B.R., Baer, S.G., Bello, N.M., *et al.* (2022). Reciprocal transplant gardens as gold standard to detect local adaptation in grassland species: New opportunities moving into the 21st century. *Journal of Ecology*, 110, 1054–1071.

Kawecki, T.J. & Ebert, D. (2004). Conceptual issues in local adaptation. *Ecol Lett*, 7, 1225–1241.

Kenzo, T., Yoneda, R., Azani, M.A. & Majid, N.M. (2008). Changes in leaf water use after removal of leaf lower surface hairs on Mallotus macrostachyus (Euphorbiaceae) in a tropical secondary forest in Malaysia. *Journal of Forest Research*, 13, 137–142.

Kitayama, K., Pattison, R., Cordell, S., Webb, D. & Mueller-Dombois. (1997). Ecological and Genetic Implications of Foliar Polymorphism inMetrosideros polymorphaGaud. (Myrtaceae) in a Habitat Matrix on Mauna Loa, Hawaii. *Ann Bot*, 80, 491–497.

Lankau, R.A. & Keymer, D.P. (2018). Simultaneous adaptation and maladaptation of tree populations to local rhizosphere microbial communities at different taxonomic scales. *New Phytologist*, 217, 1267–1278.

Lawson, T. & Blatt, M.R. (2014). Stomatal Size, Speed, and Responsiveness Impact on Photosynthesis and Water Use Efficiency. *Plant Physiology*.

Legrand, D., Cote, J., Fronhofer, E.A., Holt, R.D., Ronce, O., Schtickzelle, N., *et al.* (2017). Eco‐evolutionary dynamics in fragmented landscapes. *Ecography*, 40, 9–25.

Leimu, R. & Fischer, M. (2008). A Meta-Analysis of Local Adaptation in Plants. *PLoS ONE*, 3, e4010.

Liu, P.-C., Peacock, W.J., Wang, L., Furbank, R., Larkum, A. & Dennis, E.S. (2020). Leaf growth in early development is key to biomass heterosis in Arabidopsis. *J Exp Bot*, 71, 2439–2450.

Lortie, C.J. & Hierro, J.L. (2022). A synthesis of local adaptation to climate through reciprocal common gardens. *Journal of Ecology*, 110, 1015–1021.

Malíková, L., Latzel, V., Šmilauer, P. & Klimešová, J. (2016). Local adaptation of annual weed populations to habitats differing in disturbance regime. *Evol Ecol*, 30, 861–876.

Maricle, B.R., Koteyeva, N.K., Voznesenskaya, E.V., Thomasson, J.R. & Edwards, G.E. (2009). Diversity in leaf anatomy, and stomatal distribution and conductance, between salt marsh and freshwater species in the C_4_ genus *Spartina* (Poaceae). *New Phytologist*, 184, 216–233.

Marks, C.O. & Lechowicz, M.J. (2006). Alternative designs and the evolution of functional diversity. *Am Nat*, 167, 55–66.

Martin, R.E. & Asner, G.P. (2009). Leaf Chemical and Optical Properties of *Metrosideros polymorpha* Across Environmental Gradients in Hawaii. *Biotropica*, 41, 292–301.

Martin, R.E., Asner, G.P. & Sack, L. (2007). Genetic variation in leaf pigment, optical and photosynthetic function among diverse phenotypes of Metrosideros polymorpha grown in a common garden. *Oecologia*, 151, 387–400.

Martínez-Sancho, E., Rellstab, C., Fonti, P., Benito Garzón, M., Bigler, C., Miranda, J.C., *et al.* (2025). Genetic and plastic effects on trait variability in two major tree species: Insights from common garden experiments across Europe. *For Ecol Manag*, 597, 123126.

Mátyás, C. (1994). Climatic adaptation of trees: rediscovering provenance tests. *Euphytica*, 92, 45–54.

Matyas, C. (1994). Modeling climate change effects with provenance test data. *Tree Physiol*, 14, 797–804.

McElwain, J.C., Yiotis, C. & Lawson, T. (2016). Using modern plant trait relationships between observed and theoretical maximum stomatal conductance and vein density to examine patterns of plant macroevolution. *New Phytol*.

Meng, Z., Bailey‐Bale, J., Milner, S., Shi, P., Muchero, W., Chen, J., *et al.* (2025). Climate adaptation in *Populus trichocarpa* : key adaptive loci identified for stomata and leaf traits. *New Phytol*, 247, 2647–2664.

Ochoa, M.E., Henry, C., John, G., Medeiros, C., Pan, R., Scoffoni, C., *et al.* (2024). Pinpointing the causal influences of stomatal anatomy and behavior on minimum, operational, and maximum leaf surface conductance. *Plant Physiol*.

Ostrowsky, L., Rea, L., Hernández-Leal, M., Mohn, R., Garner, M., Worcester, L., *et al.* (2026). Evidence for adaptive variation in Quercus macrocarpa (L.) leaf morphology from a reciprocal transplant experiment across a latitudinal gradient. *For Ecol Manag*, 600, 123292.

Pan, T., Britton, T.G., Schrader, J., Sumner, E., Nicolle, D., Choat, B., *et al.* (2025). Adaptation in Wood Anatomical Traits to Temperature and Precipitation—A Common Garden Study. *Plant Cell Environ*, 48, 6016–6031.

Quero, J.L., Villar, R., Marañón, T., Zamora, R., Vega, D. & Sack, L. (2008). Relating leaf photosynthetic rate to whole-plant growth: drought and shade effects on seedlings of four Quercus species. *Functional Plant Biology*, 35, 725–737.

Ramírez-Valiente, J.A. & Cavender-Bares, J. (2017). Evolutionary trade-offs between drought resistance mechanisms across a precipitation gradient in a seasonally dry tropical oak (*Quercus oleoides*). *Tree Physiol*, 37, 889–901.

Ramirez-Valiente, J.A., Sanchez-Gomez, D., Aranda, I. & Valladares, F. (2010). Phenotypic plasticity and local adaptation in leaf ecophysiological traits of 13 contrasting cork oak populations under different water availabilities. *Tree Physiology*, 30, 618–627.

Ramírez‐Valiente, J.A., Santos Del Blanco, L., Alía, R., Robledo‐Arnuncio, J.J. & Climent, J. (2022). Adaptation of Mediterranean forest species to climate: Lessons from common garden experiments. *J Ecol*, 110, 1022–1042.

Roth-Nebelsick, A. (2007). Computer-based Studies of Diffusion through Stomata of Different Architecture. *Annals of Botany*, 100, 23–32.

Rutter, M.T. & Fenster, C.B. (2007). Testing for Adaptation to Climate in Arabidopsis thaliana : A Calibrated Common Garden Approach. *Annals of Botany*, 99, 529–536.

Sack, L. & Buckley, T.N. (2016). The developmental basis of stomatal density and flux. *Plant Physiol*.

Schierenbeck, K.A. (2017). Population-level genetic variation and climate change in a biodiversity hotspot. *Ann Bot*, 119, 215–228.

Schwinning, S., Lortie, C.J., Esque, T.C. & DeFalco, L.A. (2022). What common‐garden experiments tell us about climate responses in plants. *Journal of Ecology*, 110, 986–996.

Scoffoni, C., Chatelet, D.S., Pasquet-kok, J., Rawls, M., Donoghue, M.J., Edwards, E.J., *et al.* (2016). Hydraulic basis for the evolution of photosynthetic productivity. *Nat Plants*, 2, 16072.

Simonet, G. (2010). The concept of adaptation: interdisciplinary scope and involvement in climate change. *Sapiens*, 3.

Skelton, R.P., Anderegg, L.D.L., Diaz, J., Kling, M.M., Papper, P., Lamarque, L.J., *et al.* (2021). Evolutionary relationships between drought-related traits and climate shape large hydraulic safety margins in western North American oaks. *Proc Natl Acad Sci USA*, 118, e2008987118.

Smith, D.D., Adams, M.A., Salvi, A.M., Krieg, C.P., Ané, C., McCulloh, K.A., *et al.* (2023). Ecophysiological adaptations shape distributions of closely related trees along a climatic moisture gradient. *Nat Commun*, 14, 7173.

Sork, V.L. (2017). Genomic Studies of Local Adaptation in Natural Plant Populations. *J Hered*, 109, 3–15.

Sork, V.L., Aitken, S.N., Dyer, R.J., Eckert, A.J., Legendre, P. & Neale, D.B. (2013). Putting the landscape into the genomics of trees: approaches for understanding local adaptation and population responses to changing climate. *Tree Genetics & Genomes*, 9, 901–911.

Taylor, S.H., Franks, P.J., Hulme, S.P., Spriggs, E., Christin, P.A., Edwards, E.J., *et al.* (2012). Photosynthetic pathway and ecological adaptation explain stomatal trait diversity amongst grasses. *New Phytologist*, 193, 387–396.

Tsvuura, Z. & Ward, D. (2022). Does a reciprocal transplant experiment of neighboring Vachellia karroo populations demonstrate local adaptation? *South African Journal of Botany*, 144, 316–324.

Tudor, E.P., Lewandrowski, W., Krauss, S. & Veneklaas, E.J. (2024). Local adaptation to climate inferred from intraspecific variation in plant functional traits along a latitudinal gradient. *Conserv Physiol*, 12, coae018.

Wang, R., Yu, G., He, N., Wang, Q., Zhao, N., Xu, Z., *et al.* (2015). Latitudinal variation of leaf stomatal traits from species to community level in forests: linkage with ecosystem productivity. *Sci Rep*.

Wang, S. & Zhou, D. (2021). Morphological canalization, integration, and plasticity in response to population density in *Abutilon theophrasti* : Influences of soil conditions and growth stages. *Ecology and Evolution*, 11, 11945–11959.

Weraduwage, S.M., Chen, J., Anozie, F.C., Morales, A., Weise, S.E. & Sharkey, T.D. (2015). The relationship between leaf area growth and biomass accumulation in Arabidopsis thaliana. *Front. Plant Sci.*, 6.

Wilczek, A.M., Cooper, M.D., Korves, T.M. & Schmitt, J. (2014a). Lagging adaptation to warming climate in *Arabidopsis thaliana*. *Proc. Natl. Acad. Sci. U.S.A.*, 111, 7906–7913.

Wilczek, A.M., Cooper, M.D., Korves, T.M. & Schmitt, J. (2014b). Lagging adaptation to warming climate in *Arabidopsis thaliana*. *Proc Natl Acad Sci USA*, 111, 7906–7913.

Yang, J., Cao, M. & Swenson, N.G. (2018). Why functional traits do not predict tree demographic rates. *Trends Ecol Evol*, 33, 326–336.

Yavari, N., Tripathi, R., Wu, B.-S., MacPherson, S., Singh, J. & Lefsrud, M. (2021). The effect of light quality on plant physiology, photosynthetic, and stress response in Arabidopsis thaliana leaves. *PLoS ONE*, 16, e0247380.

Yu, J., Pressoir, G., Briggs, W.H., Vroh Bi, I., Yamasaki, M., Doebley, J.F., *et al.* (2006). A unified mixed-model method for association mapping that accounts for multiple levels of relatedness. *Nat Genet*, 38, 203–208.
